# Changes in the western flank of the North Atlantic subtropical high since 1140 CE: Extremes, drivers, and hydroclimatic patterns

**DOI:** 10.1126/sciadv.adr5065

**Published:** 2025-04-16

**Authors:** Joshua C. Bregy, Justin T. Maxwell, Scott M. Robeson, Grant L. Harley, Valerie Trouet

**Affiliations:** ^1^ Department of Environmental Engineering and Earth Sciences, Clemson University, Clemson, SC, USA.; ^2^ Glenn Department of Civil Engineering, Clemson University, Clemson, SC, USA.; ^3^ Department of Geography, Indiana University, Bloomington, IN, USA.; ^4^ Department of Earth and Spatial Sciences, University of Idaho, Moscow, ID, USA.; ^5^ Laboratory of Tree-Ring Research, University of Arizona, Tucson, AZ, USA.; ^6^ Belgian Climate Centre, Brussels, Belgium.

## Abstract

Summer circulation and moisture patterns in the Southeast United States are controlled by the position of the North Atlantic subtropical high. In a warming climate, the subtropical high is projected to strengthen and expand west, but there remains uncertainty regarding its variability and linkages to natural drivers. Here, we use a tree-ring network across the Southeast United States to reconstruct the relative intensity of the pressure gradient across the subtropical high’s western flank over the past 870 years. Variations in the flank’s position and the pressure gradient have been a major driver of the hydroclimate—including creating a Southeast-Caribbean moisture dipole—since 1140 CE. We document a significant increase in flank positional variability since 1900 CE, with westward migrations becoming more extreme. Likewise, major volcanic eruptions cause a multiyear period of westward positioning, leading to distinct regional moisture gradients. Our record highlights important changes in flank behavior, which has important implications for water resource management in a warming world.

## Teaser

Eight centuries of Bermuda high patterns show a 50% increase in western flank variability starting in 1900 CE.

## INTRODUCTION

**Fig. 1.**
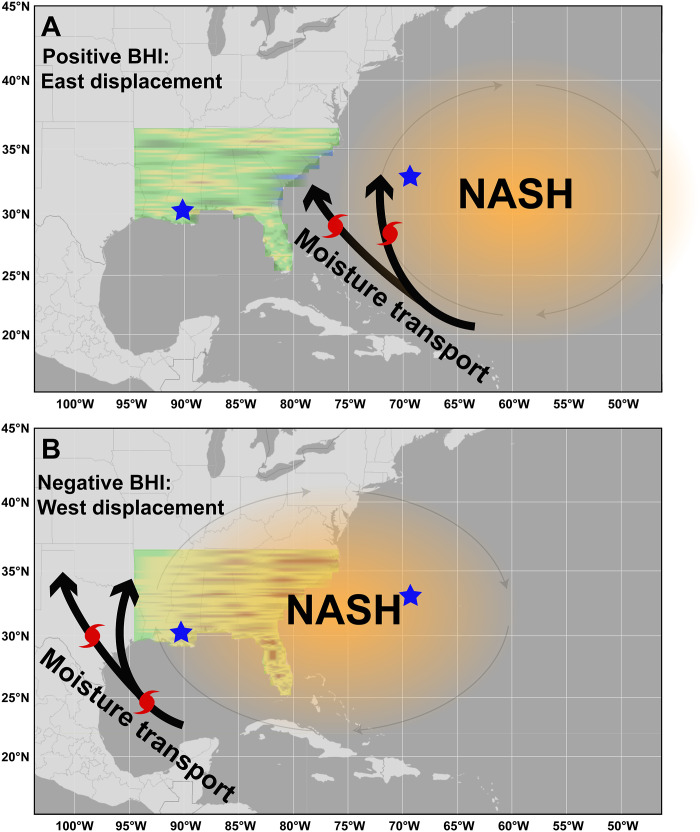
East-west positioning of the NASH. Conceptual diagram of moisture patterns in the southeastern US associated with the distinct positions of the NASH. Stars represent Bermuda (eastern star) and New Orleans, Louisiana, US (western star), which are the locations of the monthly mean sea level pressure (MSLP) time series that are used to calculate the BHI. Background colors in the southeastern US are used to show moisture patterns, with green and brown indicating wet and dry conditions, respectively. (**A**) Positive BHI values indicate an eastward positioning of the NASH and its western flank. This gives rise to variable spatial and interannual moisture patterns, although increased moisture can be seen on the east coast depending on the location of the flank. (**B**) Negative BHI values indicate a westward positioning of the NASH and its western flank. When the NASH is positioned to the northwest, drier conditions prevail across the Southeast US.

**Fig. 2.**
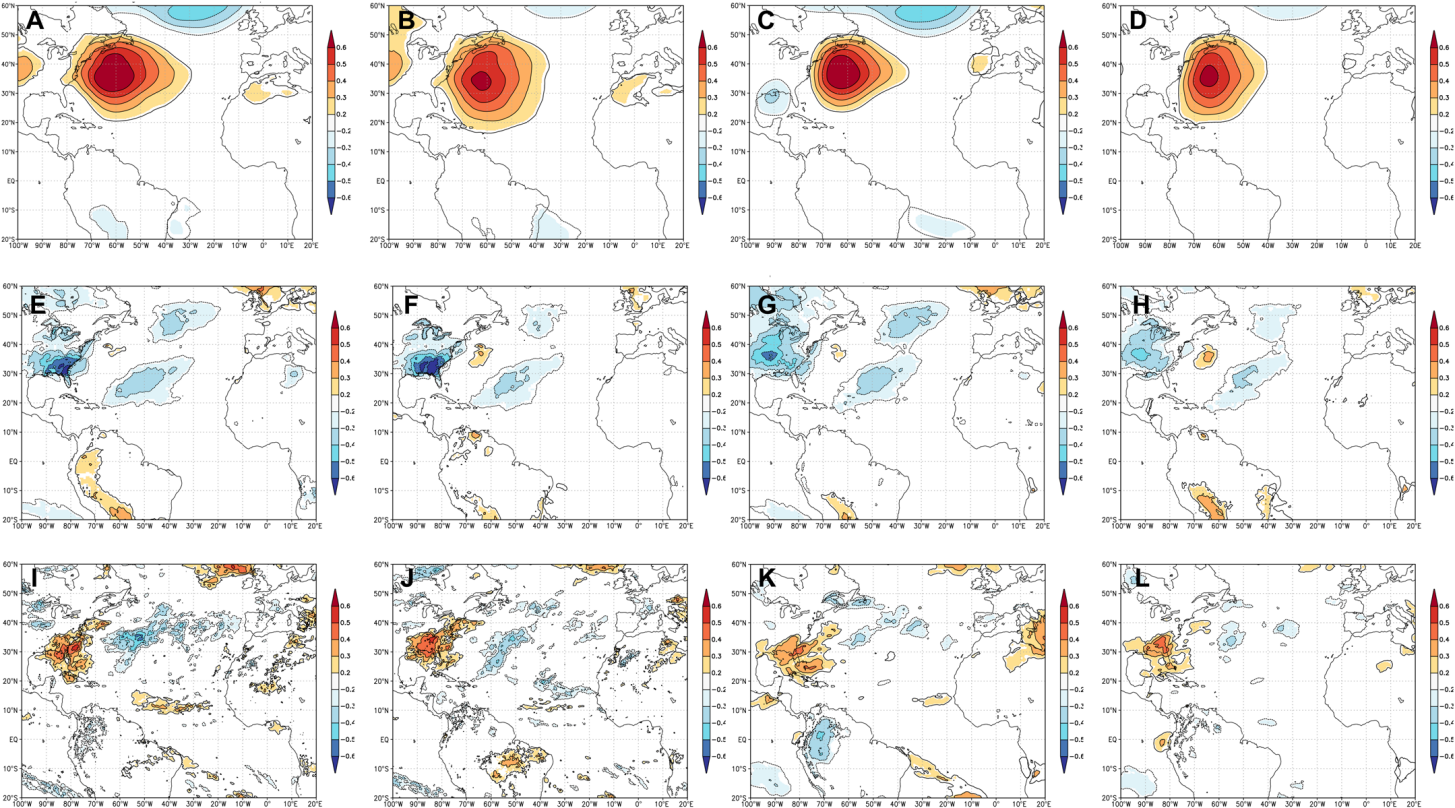
Field correlations between the BHI and ERA5 variables. Field correlations between instrumental (**A**, **C**, **E**, **G**, **I**, and **K**) and reconstructed (**B**, **D**, **F**, **H**, **J**, and **L**) BHI and different European Centre for Medium-Range Weather Forecasting Reanalysis v5 (ERA5) variable averages over May to July (MJJ; 1950–2009 CE). (A) Correlations between instrumental BHI and MSLP (in millibars) from the ERA5 dataset. Only areas with significant correlations (*P* < 0.1) are contoured. (B) Same as in (A) but using the reconstructed BHI. (C) Correlations between instrumental BHI and 850-hPa geopotential heights (in meters) from the ERA5 dataset. Only areas with significant correlations (*P* < 0.1) are contoured. (D) Same as in (C) but using the reconstructed BHI. (G) Correlations between instrumental BHI and minimum surface temperature (in degrees Celsius) from the ERA5 dataset. Only areas with significant correlations (*P* < 0.1) are contoured. (H) Same as in (G) but using the reconstructed BHI. (I) Correlations between instrumental BHI and total precipitation (in millimeters) from the ERA5 dataset. Only areas with significant correlations (*P* < 0.1) are contoured. (J) Same as in (I) but using the reconstructed BHI. (K) Correlations between instrumental BHI and 850-hPa relative humidity from the ERA5 dataset. Only areas with significant correlations (*P* < 0.1) are contoured. (L) Same as in (K) but using the reconstructed BHI. All analyses and figures were completed using KNMI Climate Explorer (*73*).

Subtropical highs are semipermanent anticyclones over oceans that connect the tropical and midlatitude wind belts and influence moisture transport in the subtropics and midlatitudes (*1*, *2*). Summer precipitation variability in the southeastern United States (US) shows a large interannual variation (*3*, *4*) and is strongly influenced by moisture transport via the North Atlantic subtropical high (NASH) (*5*–*11*). The position and intensity of the NASH vary throughout the year, as its center migrates between Bermuda and the Azores during the boreal summer and winter seasons, respectively (*12**,* *13*). During the boreal summer, the circulation around its western flank (hereinafter flank) directs moisture across the southeastern US, frequently producing anomalously dry conditions when positioned to the west (Fig. 1A). Conversely, when the flank is positioned farther east during the summer, it can direct moisture into the southeastern US, especially along the east coast and southern Appalachia (Fig. 1B). Strong correlations between an index of the pressure gradient across the flank [Bermuda high index (BHI)] and multiple climate variables [e.g., Palmer drought severity index (PDSI), standardized precipitation evapotranspiration index (SPEI), surface temperatures, zonal and meridional winds, cloud cover, insolation, etc.] are widespread across eastern North America and the western North Atlantic Ocean (Fig. 2 and figs. S4 to S8) (*5*, *6*). Similar patterns also exist when using 850-hPa geopotential heights to represent the flank (*7*–*10*). These patterns demonstrate that the flank is an important mechanism for regional climate variability.

Climate projections indicate that the NASH will strengthen and exhibit more frequent periods of western migration toward the southeastern US in response to enhanced land-sea thermal contrast driven by anthropogenic warming (*14*, *15*). This has important implications for extremes in the hydroclimate (i.e., pluvials, droughts, and tropical cyclones), which are projected to intensify in response to anthropogenic greenhouse gas forcing (*16*). In addition, the NASH acts as a dynamic control on precipitation in the southeastern US, and, thus, the projected intensification of the former should increase summer rainfall variability in the region (*7*). The associated hydroclimatic changes can cause undue stress on water resources to meet the demands of the agricultural, industrial, and municipal sectors. For example, increased precipitation variability can lead to increased variability in crop yield, exacerbating the already high strain in place on water supply and further affecting agriculture and food supplies (*17*–*19*). However, much of what is known about the NASH comes from the short instrumental record (100 to 150 years), which has only exhibited statistically significant changes to the flank since the 1970s (*7*, *8*, *11*). Consequently, recent records only provide a snapshot of flank variability under the influence of anthropogenic greenhouse gas forcing. Thus, a longer baseline for the natural variability exhibited by the flank is needed to provide a broader context for modern variations of NASH behavior and the associated hydroclimatic changes.

Paleoclimatic records derived from tree rings can extend records of atmospheric circulation beyond the instrumental period, better establishing interdecadal variations and uncovering extremes in the climate system (*20*–*24*). While tree growth does not respond to atmospheric pressure directly, it does respond to large-scale environmental factors that drive growth (e.g., precipitation and temperature), which are functions of pressure and circulation anomalies (*21*). Moreover, the range of growth factors influenced by pressure and circulation allows for multiple species to be incorporated into a reconstruction. In essence, reconstructions of pressure or circulation anomalies integrate multiple climatic variables, making this type of reconstruction particularly useful for uncovering regional to large-scale climate patterns (Fig. 2).

Given the importance of the flank in regulating regional precipitation patterns and anomalies in the southeastern US (Fig. 2 and figs. S4 to S6) (*6*–*8*, *25*–*27*), diverse tree-ring networks are invaluable for reconstructing large-scale atmospheric circulation variability and extreme conditions, particularly during the warm season when the NASH is typically positioned at its westernmost extent (*25*). During this period, the flank exerts considerable influence on different climate features, especially precipitation (*4*, *6*–*13*), that strongly affect tree growth (*21*). Here, we provide the first quantitative reconstruction of the pressure gradient across the flank of the NASH using a comprehensive tree-ring network across the southeastern US (fig. S1 and table S1). Specifically, we reconstruct the mean May to July (MJJ) BHI, which is a standardized representation of east-west variations in the flank of the NASH that uses differences in mean sea level pressure (MSLP) between New Orleans, Louisiana, US, and Bermuda (*6*, *25*). This metric captures positional patterns in the flank driving regional hydroclimate variability. Our annually resolved, warm-season BHI record is derived from an extensive tree-ring network and extends back to 1140 CE. We use our 870-year reconstructed record of MJJ BHI to (i) examine the variability and extremes in the flank, (ii) explore the associated hydroclimatic shifts, and (iii) understand the long-term forcings driving flank variability.

## RESULTS

### Trends and variability in the western flank

**Fig. 3.**
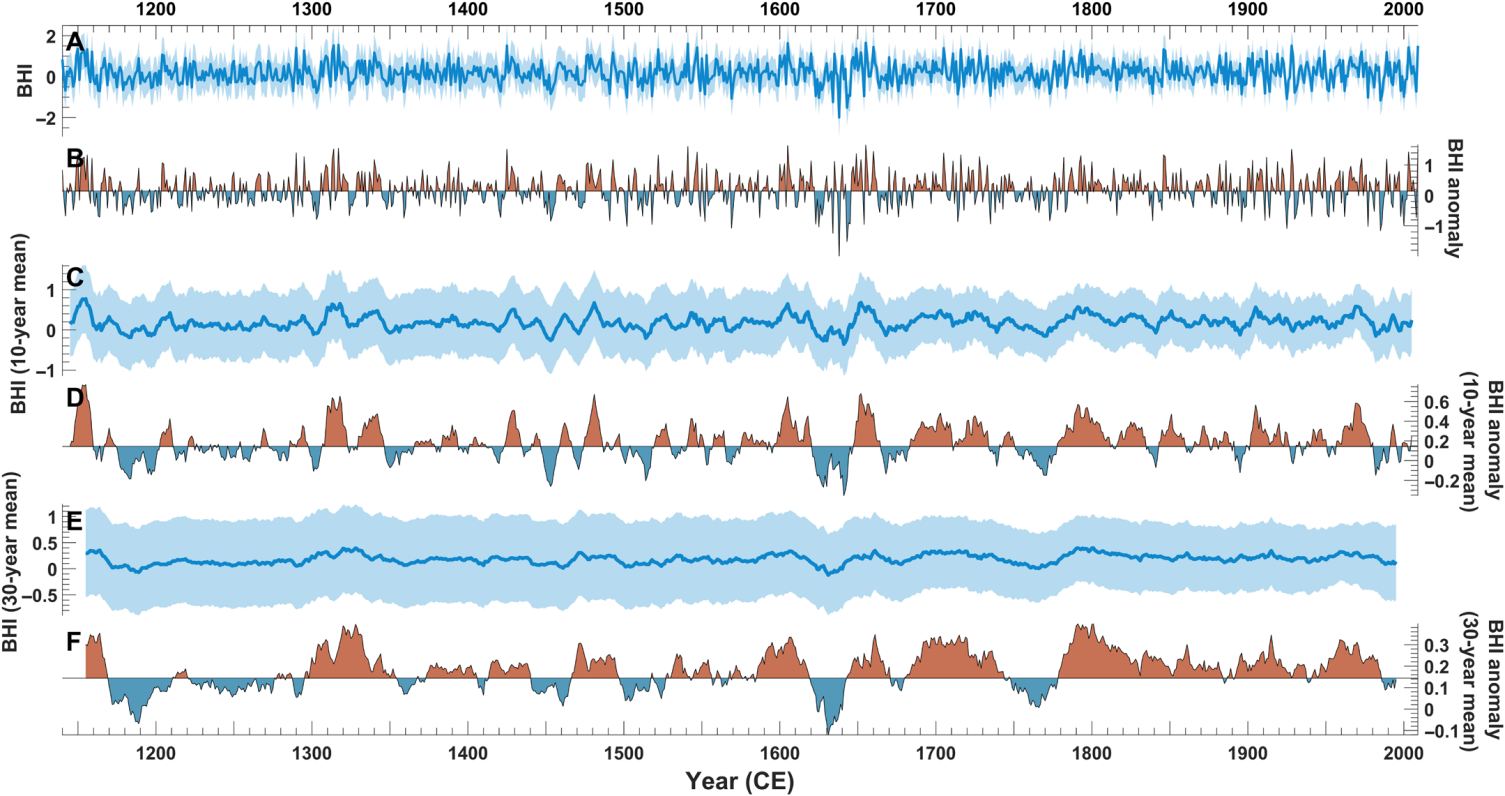
Reconstructed BHI values and anomalies relative to the 1948–2009 mean. (**A**) Annual reconstruction of BHI values from 1140 to 2009 CE. Blue shading represents lower (5th) and upper (95th) confidence bounds. (**B**) Annual BHI anomalies with respect to the mean instrumental BHI from 1948 to 2009 CE (mean BHI = 0.1440). (**C**) Ten-year moving average of BHI values from 1140 to 2009 CE. Blue shading represents lower (5th) and upper (95th) confidence bounds. (**D**) BHI anomalies using a 10-year moving average relative to the mean instrumental BHI from 1948 to 2009 CE. (**E**) Thirty-year moving average of BHI values from 1140 to 2009 CE. Blue shading represents lower (5th) and upper (95th) confidence bounds. (**F**) BHI anomalies using a 30-year moving average relative to the mean instrumental BHI from 1948 to 2009 CE.

Our reconstruction of the MJJ BHI (1140–2009 CE) explains 71% of the variance in BHI based on the first reconstruction nest (see Materials and Methods, Fig. 3, and fig. S2). This seasonal window represents the period when the NASH is at its maximum western extent (*6*, *13*, *25*), and, therefore, it exerts a strong influence on climate patterns across the Southeast US (Fig. 2 and figs. S4 to S6) (*4*, *6*–*13*, *25*–*27*). Both the instrumental and reconstructed BHI values are from similar distributions (fig. S3). Field correlations between the reconstructed BHI and the different climate variables further support the robustness of our reconstruction relative to the instrumental record in which the magnitude and spatial patterns are similar between instrumental and reconstructed BHI values (Fig. 2 and figs. S4 to S6). Furthermore, this is also seen in correlations between the European Centre for Medium-Range Weather Forecasting Reanalysis v5 data (ERA5) and both instrumental and reconstructed BHIs (Fig. 2). For example, BHI is strongly correlated with MSLP (Fig. 2, A and B) and 850-hPa geopotential heights (Fig. 2, C and D), highlighting the ability of the BHI to represent flank behavior. Likewise, strong correlations between both BHI records and temperatures (Fig. 2, E to H) and precipitation (Fig. 2, I and J) occur across the field sites, further supporting the connection between tree rings and integrated climate variables, such as circulation indices.

**Fig. 4.**
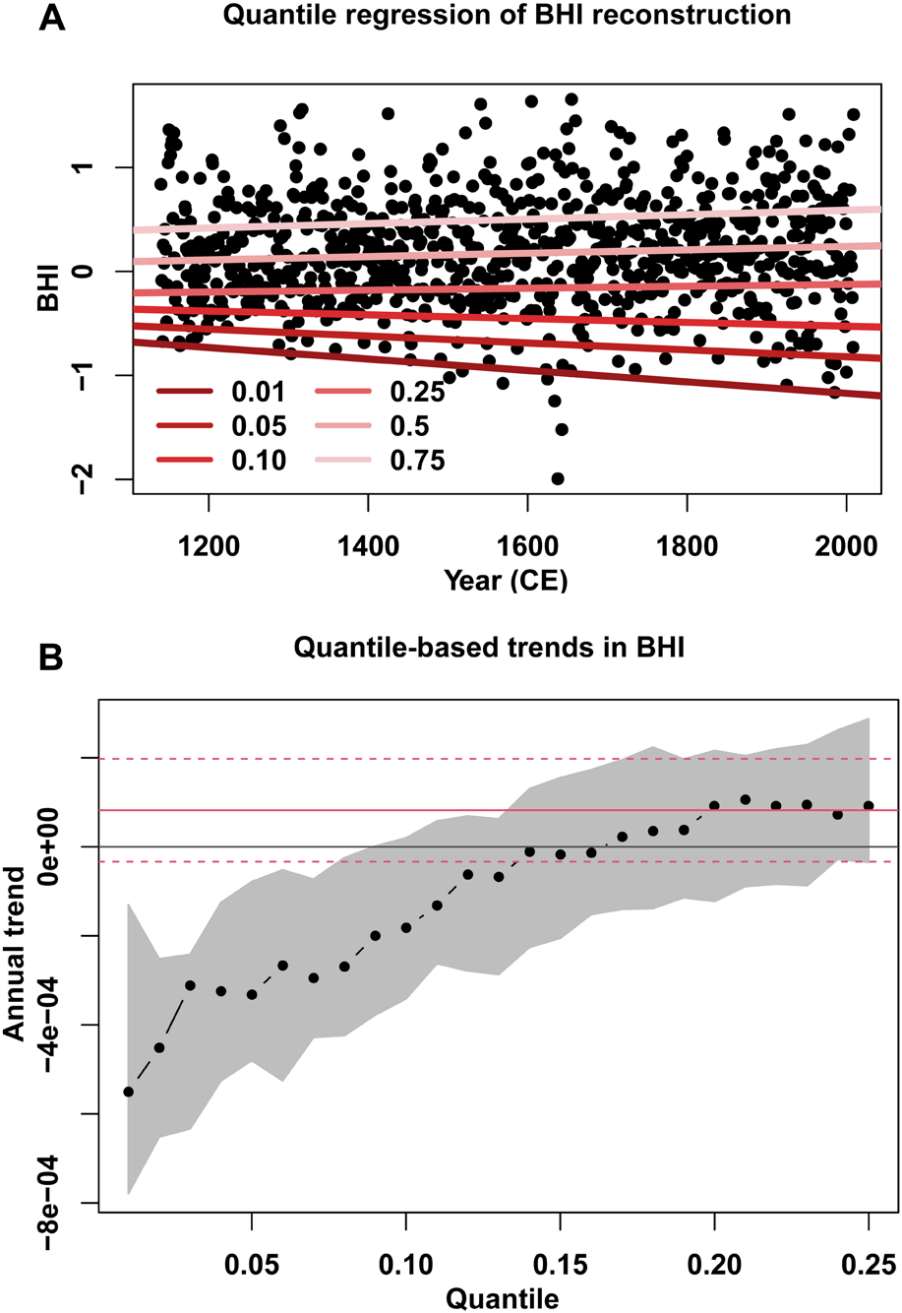
Quantile regression and trends for the BHI. (**A**) Quantile regression scatterplot for the BHI. Red-hued lines are regression lines for different quantiles. (**B**) Annual trends in the lower quantiles (≤0.25 quantile) of the BHI (unitless). Values outside of the red lines (solid and dashed) are significant at α = 0.05. Quantile regressions were performed using the R package "quantreg" (*76*).

Whether the flank has exhibited any trends in the frequency of westward excursions during the 20th and 21st centuries is hotly debated (*7*, *28*–*32*): Geopotential heights (850 hPa) indicate that the flank has migrated west since 1948 CE (*7*), while the BHI suggests an eastward migration since the late 1970s (*31*). It has been suggested that these differences stem from the different variables (see Materials and Methods) and sampling periods used (*29*). We do not see any significant annual trends in the MJJ instrumental (*P* = 0.33) or reconstructed BHI (*P* = 0.84) since 1948 CE, confirming the results from prior work for the same period [1948–2009 CE (*29*)]. Despite the absence of an annual trend in the mean BHI, quantile regression shows distinct negative trends in the MJJ BHI at or below the 10th percentile (BHI = −0.4225; Fig. 4) during the past 870 years, suggesting a weakening of the pressure gradient along the flank. Therefore, the intensity of extreme westward incursions of the flank has been increasing, coincident with a shift in the mean state, a finding that agrees with the changes expected under warming. More frequent westward shifts have increased precipitation variability in the southeastern US, which is documented in instrumental records (*7*) and is expected to continue increasing in response to flank shifts under current warming trends (*7*, *9*, *27*).

Another critical change seen in the BHI reconstruction is an increase in the variability of the flank’s east-west position during the 20th and 21st centuries. Despite prior studies presenting differing trends in the flank, the data generally show an increase in variance since 1948 CE (*7*, *8*, *14*, *15*, *28*–*32*). In our record, a significant increase in variability in the BHI occurs during the 20th century (1900–2009 CE; figs. S9 and S10), a period marked by the greatest increase in anthropogenic greenhouse gas radiative forcing (*33*, *34*), compared to the end of the Medieval Climate Anomaly (MCA) (~1140–1250 CE; *P* = 0.011, *F* statistic = 1.6, *s*^2^_20thC_ = 0.38, *s*^2^_MCA_ = 0.24) (*35*) and the Little Ice Age (LIA) (~1300–1850 CE; *P* = 0.0088, *F* statistic = 1.4, *s*^2^_LIA_ = 0.27) (*35*). Overall, there is a 50% increase in BHI variability during the 20th century compared to the entire preindustrial period (*P* = 0.0033, *F* statistic = 1.5, *s*^2^_preindustrial_ = 0.26). The recent increase seen in our record indicates that the summer flank position is becoming increasingly variable, along with a shift in the mean state in the BHI, in response to ongoing warming (figs. S9 and S10) (*7*, *8*, *14*, *15*, *36*). Therefore, in the context of the ongoing warming trend, we expect the flank of the NASH to behave similarly to projections from the Coupled Model Intercomparison Projects phases 3 and 5: increased interannual positional variability and frequent anomalous westward migrations. This would lead to increased variance in summer precipitation, including its spatial distribution, intensity, and interannual variability across the southeastern US and the Caribbean (*7*–*10*, *14*, *15*, *25*, *27*) while also influencing tropical cyclone tracks and precipitation patterns (*37*–*39*) and streamflow (*40*, *41*).

### Hydroclimatic role of the western flank

**Fig. 5.**
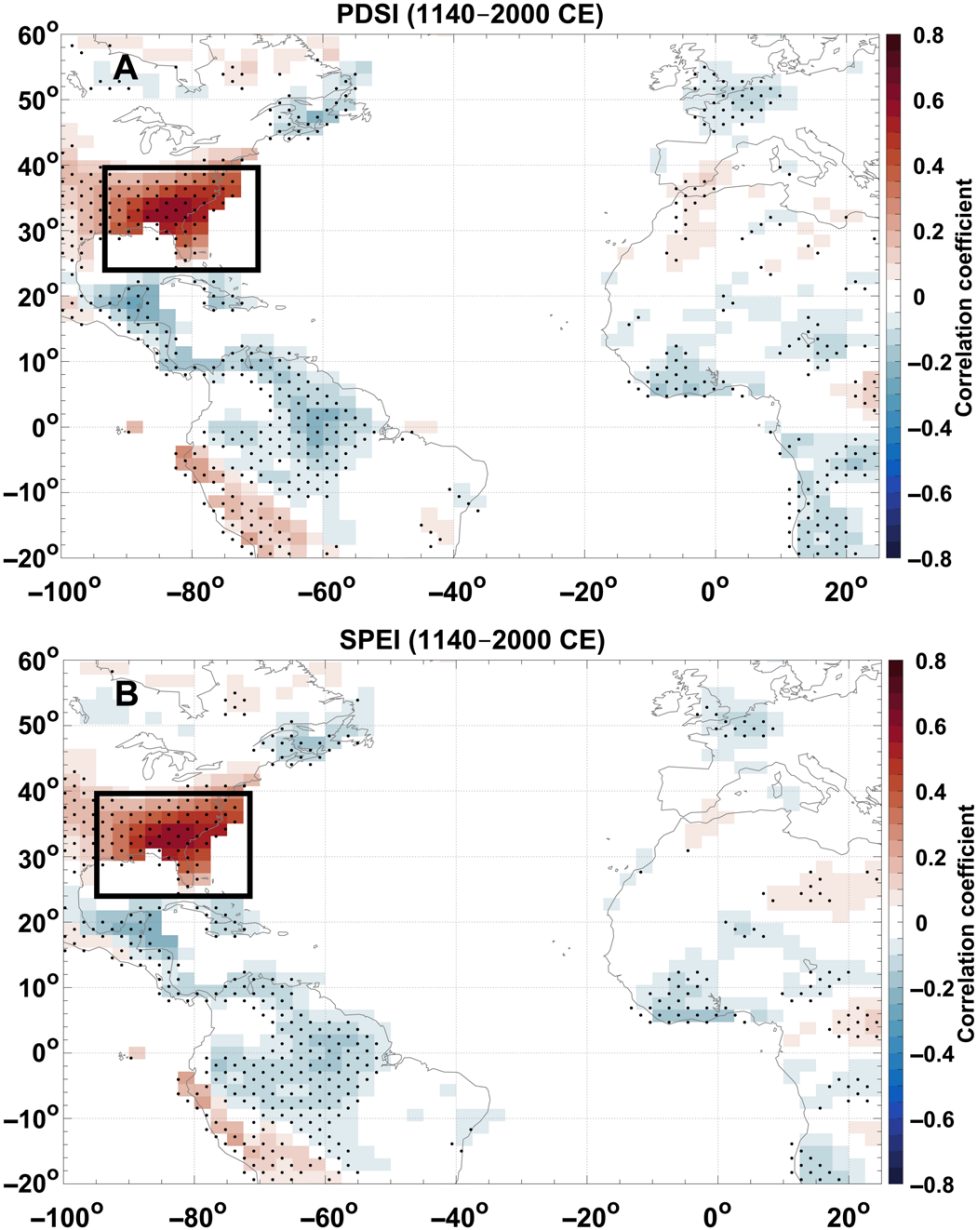
Correlations between the BHI and moisture indices. Field correlation maps between the reconstructed BHI and moisture indices derived from the PHYDA product. The black box indicates the region where the chronologies are located. (**A**) Correlation coefficients between the BHI and PDSI from 1140 to 2000 CE. Stippled regions indicate significant correlations (*P* ≤ 0.05). (**B**) Same as in (A) but between BHI and SPEI.

Modeled PDSI and SPEI values from the Paleo Hydrodynamics Data Assimilation (PHYDA) product (*42*) are moderately to strongly correlated with reconstructed BHI (0.3 ≤ *r* < 0.7; *P* < 0.05) across most of the region, especially in the eastern and east-central half of the Southeast US (Fig. 5). As the flank moves west (negative BHI), dry conditions (negative PDSI and SPEI) manifest across the Southeast US owing to evaporation rates exceeding precipitation rates in the region. Elsewhere in the contiguous US, we see weak to moderate correlations between the BHI and drought metrics. Significant inverse correlations (−0.4 < *r* ≤ −0.1; *P* < 0.05) exist across much of the Caribbean and Central America (Fig. 5). This relationship persists over decadal timescales, with the same regions exhibiting significant correlations of similar magnitude between the 5-year averages of the BHI, PDSI, and SPEI, albeit at a weaker magnitude for both positive (0.2 ≤ *r* ≤ 0.5; *P* < 0.05) and negative correlations (−0.3 ≤ *r* ≤ −0.1; *P* < 0.05; fig. S12).

The relatively wide range of correlation coefficients, including maximum values of ~0.7, may indicate that there is also a transition point between the position of the flank and moisture availability in a region. As the flank moves west, locations beneath the flank will undergo drying, whereas regions to the west will experience increased moisture. However, as the flank continues migrating west, areas that previously received moisture will also undergo drying. Conversely, if the flank moves east, then the areas within the climatological domain of the flank will be too distant from the flank; therefore, moisture in these regions is controlled by other factors, synoptic or smaller.

The positive correlation reflects the positionally dependent role of the flank in directing moisture to different areas across the region (Fig. 1), creating a moisture dipole between the southeastern US and the Caribbean–Central America region (Fig. 5 and figs. S13 to S16). Prior work has indicated the presence of this dipole in modern records (*27*) and an inter-Caribbean dipole during the middle to late Holocene (*43*). When the flank is closer to its mean position or shifted to the east, moisture is directed into the southern Appalachian corridor and east coast. Conversely, as the flank migrates west, this region and much of the southeastern US can dry, as high pressure is positioned over the area. In this case, moisture is directed into other regions, such as the Caribbean and the Yucatán Peninsula, thereby contributing to a moisture dipole.

### Volcanic forcing of the western flank

**Fig. 6.**
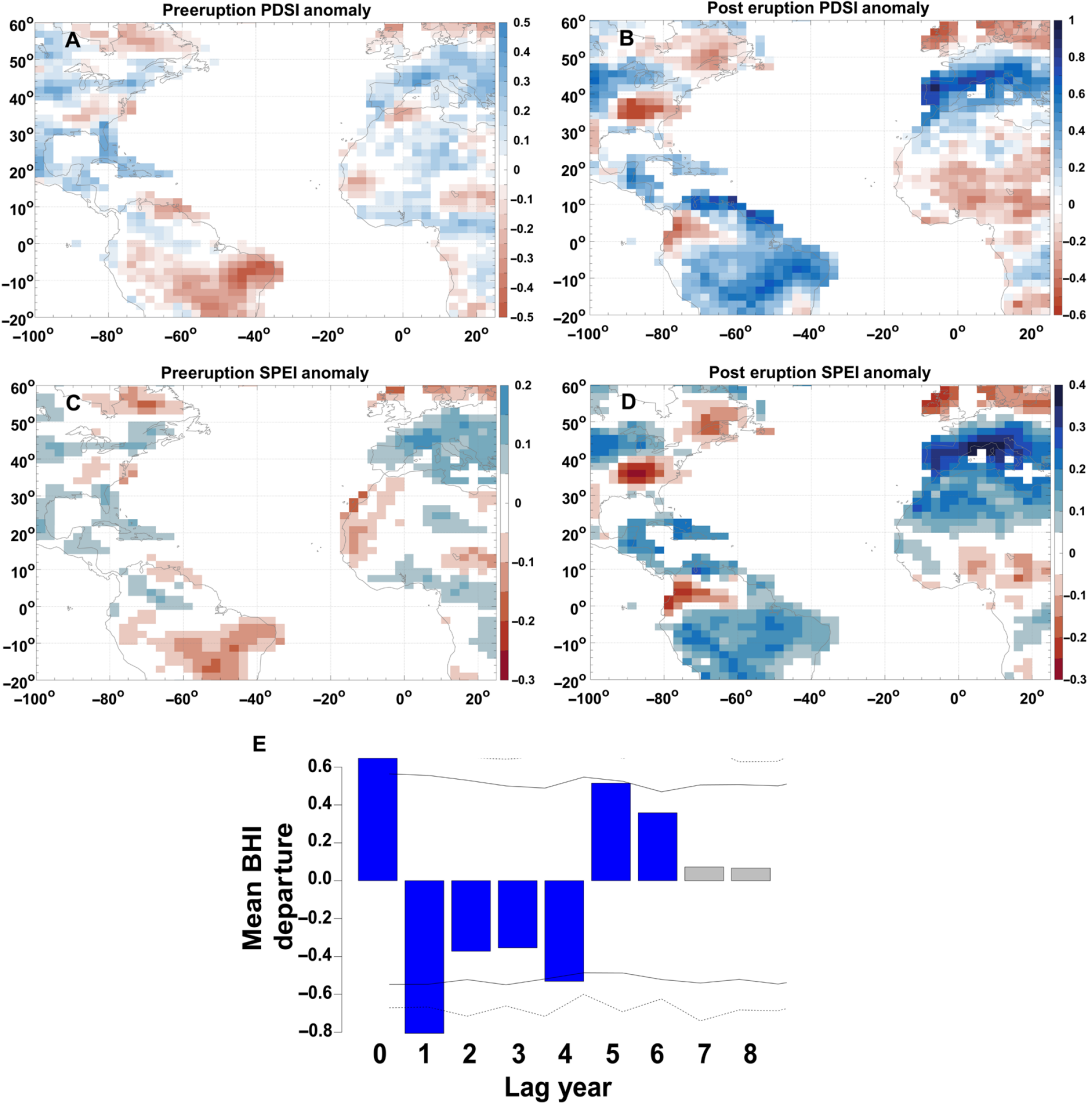
Volcanic eruption effects on moisture and the BHI. Mean pre- and posteruption anomalies seen in moisture indices from the PHYDA product and SEA results. (**A**) Mean preeruption (*t*_0_ – 5 to *t*_0_ – 1) PDSI anomalies relative to the study period mean (1140–2000 CE). (**B**) Mean posteruption (*t*_0_ + 1 to *t*_0_ + 5) PDSI anomalies relative to the study period mean (1140–2000 CE). (**C**) Same as in (A) but using SPEI. (**D**) Same as in (B) but using the SPEI. Refer to table S2 for a list of volcanoes used and volcano years. (**E**) Mean BHI departure relative to the period of record mean (+0.6) following explosive tropical volcanic eruptions. Fourteen eruptions were included in the SEA (table S2). Blue shading indicates significance at *a* = 0.1, whereas the solid and dashed lines represent significance at *a* = 0.05 and *a* = 0.01, respectively. The lag year is the year following an eruption. SEA was done using the sea function in the R package dplr (*81*).

Since 1850 CE, there have only been three major volcanic eruptions that meet our inclusion criteria [volcanic explosivity index (VEI) ≥ 5; see Materials and Methods and table S2] (*44*, *45*), and the observed influence of eruptions of flank behavior is therefore limited. However, using our 870-year reconstruction, we demonstrate that major volcanic eruptions influence the flank of the NASH (Fig. 6 and table S3). In the eruption year (*t*_0_), the flank undergoes a significant eastward migration (mean BHI departure, +0.65; *P* = 0.005). This is followed by a westward migration of the flank in the subsequent year (*t*_0_ + 1; mean BHI departure, −0.81; *P* = 0.001), where the summer flank tends to remain during the next 3 years (*t*_0_ + 2 – *t*_0_ + 4; mean BHI departure, −0.35 to −0.53; *P* = 0.019 to 0.091) before lastly shifting eastward at years 5 and 6 (*t*_0_ + 5: mean BHI departure, 0.52; *P* = 0.022; *t*_0_ + 6: mean BHI departure, 0.36; *P* = 0.083). Beyond this, the mean departures are not significant. While we see significant values, albeit at different thresholds, for up to 6 years following an eruption, the effects of radiative forcing from volcanoes are generally limited to the first few years following an eruption. Consequently, the latter years of our superposed epoch analysis (SEA) should be interpreted with some caution.

We limit our analyses to 5 years posteruption, as the responses of the Atlantic multidecadal oscillation and the intertropical convergence zone (ITCZ) in the tropical Atlantic and Africa occur during the first 5 years following an eruption (*45*). Following an explosive eruption, declines in moisture are seen across much of the southeastern US (Fig. 6 and figs. S17 to S20), indicative of a westward migration of the flank. Despite this positioning, cooling typically occurs in this region following an eruption (figs. S21 to S23), indicating that cooling from eruptions may supersede warming incurred from the NASH. Beyond the lower probability of westward migrations after *t*_0_ + 1 (Fig. 6E and table S3), preeruption conditions will influence the response of the climate system to volcanic eruptions (*46*, *47*), which could explain instances during which climatic changes are not representative of westward migrations. Nevertheless, we see a distinct hydroclimatic signal that is indicative of these east-west migrations following eruptions (Fig. 6), regardless of whether the anomalies are relative to preeruption conditions (figs. S18 and S20) or the entire record (figs. S17 and S19).

## DISCUSSION

Using our 870-year BHI reconstruction, we can robustly reconstruct and characterize the summer patterns exhibited by the flank. We identified three major features of the position of the flank of the NASH. First, we see increased variability in flank positioning during the 20th century compared to the preindustrial period, including the MCA and LIA. This variability is driven by a distinct trend toward more extreme (≤10th percentile) westward positioning of the flank. These findings are consistent with the projected changes to the NASH in response to warming, namely, increasing variance in and extent of westward migrations (*14*, *36*).

Second, the flank has acted as a critical driver of hydroclimate variability in the Caribbean and southeastern US over the past millennium, with its position giving rise to a moisture dipole between the regions. This dipole has been identified in both 20th-century instrumental records (*27*) and during the mid- to late Holocene (*43*), and is frequently linked to the position of the NASH. This dipole is present when the flank is positioned farther west (negative BHI; Fig. 6, B and D, and figs. S13 to S16). The NASH is unlikely the sole source of this dipole, especially considering the range of factors driving precipitation and evapotranspiration variability in the Southeast US, Central America, and the Caribbean (*27*, *43*). While we found no significant correlations between the flank and ITCZ latitude, previous work suggests that both the NASH and the position of the ITCZ have acted in concert to control centennial-scale moisture variability in the region (*43*). However, when controlling for the latitude of the ITCZ, we see no change in the correlations—neither distribution, magnitude, nor significance—between BHI and PHYDA outputs. Consequently, the role of the ITCZ might not be as strong at annual timescales and during early summer (MJJ) because the NASH tends to dominate lower tropospheric circulation across much of the western North Atlantic.

Third, we see a connection between volcanic eruptions and flank variability. Immediately following an eruption, the flank migrates east before moving west for several years. One potential mechanism for the westward migration of the flank following an eruption could be changes in the West African and North American monsoons. The formation and strengthening of the NASH during boreal summer are forced, in part, by land-sea thermal gradients in the West African monsoon domain (*1*, *48*, *49*) and the Rossby waves generated by the North American monsoon through a vorticity dipole aloft (*1*, *48*). Prior work demonstrates that West Africa undergoes prolonged drying following tropical eruptions, suggesting a weakening of the West African monsoon in response to a net decline in downward shortwave radiation (*45*). PHYDA temperatures do not show cooling in the West African monsoon region following an eruption (figs. S21 to S23) (*50*), but there is drying throughout West Africa (Fig. 6 and figs. S17 to S20) (*45*), indicative of a weakening of the West African monsoon. Similarly, drying in the North American monsoon domain is observed following tropical eruptions (*45*), suggesting a weakening of the monsoon that leads to a decline in Rossby wave formation (*48*). Because both monsoons are weakening, including the subsequent reduction in thermal gradients and Rossby waves, the NASH may undergo a period of weakening, briefly shifting east, following a tropical volcanic eruption. Further, the westward migration of the flank in the years after an eruption likely reflects the reestablishment of the land-sea temperature difference, as the land temperatures return to near-normal levels, while ocean temperatures remain cooler.

An alternative mechanism for our reconstructed variations in BHI could be cross-equatorial advection of cold air from the Southern Hemisphere (*51*). Equatorward intrusions of extratropical cold air in the Southern Hemisphere weaken temperature gradients in the lower troposphere, with the resulting shallow tropospheric circulation increasing the pressure and northward return flow below 500 hPa in the subtropical North Atlantic (*51*). Consequently, this leads to a strengthening and expansion of the NASH, particularly along its flank (*51*). Variations in interhemispheric exchanges have been identified following explosive volcanic eruptions (*52*, *53*), and the influence of cross-equatorial advection on the NASH during boreal summer (*52*) may, therefore, increase following explosive tropical eruptions, especially if there is a widespread breakdown of Northern Hemisphere monsoons. Ultimately, the controls on NASH behavior following an eruption are complex, and the proposed mechanisms likely work together to drive these changes, particularly by modifying the land-sea temperature difference that is critical for the summer NASH. Therefore, future work will require incorporating paleoclimate modeling and data assimilation techniques to provide a complete understanding of the external and internal forcings driving changes in the NASH.

Ultimately, our reconstruction can be used to assess the drivers behind drought and pluvial episodes across the southeastern US and the Caribbean, including understanding changes to hydroclimatic extremes in response to external forcings. Naturally, this information is critical for tuning models to understand the changes in subtropical circulation in response to climate change better. This will provide important insight into the range of hydroclimatic shifts that are expected in the southeastern US and the Caribbean. Future work will require extending the seasonal coverage of flank reconstructions to assess interactions between the NASH and extreme precipitation sources (e.g., tropical cyclones and atmospheric rivers) and associated flooding over different timescales.

As climate change continues to strain water resources and infrastructure, our reconstruction is an important aid in developing appropriate water resource and hazard mitigation plans that lessen the economic burden of extreme events associated with warming. For example, the flank is a major influence on crop yield in the Southeast US (*17*). Given the direct relationship between precipitation and crop yield variability (*18*), agricultural productivity will likely become more volatile as precipitation patterns and evaporative demand shift in response to the increasingly variable behavior of the flank. Considering that crop model projections tend to underestimate low yields and crop failures (*54*), our reconstruction can provide important context for models to improve our understanding of crop changes and management strategies. From the perspective of hydroclimate hazards, our reconstruction can help provide critical insight into drought dynamics and tropical cyclone patterns, particularly how these hazards covary with the flank, as the climate continues warming. In turn, this may inform strategies and policies aimed at mitigating the effects of these hazards on vulnerable communities and economic sectors. To that end, while outside the scope of this study, additional work exploring the relationship between BHI and NASH intensity in dynamic models is necessary to evaluate how well flank variability is represented by models and to provide straightforward projections of changes to the flank for different economic and environmental sectors. Together, our results demonstrate substantial changes to the flank of the NASH over the past millennium, including an unprecedented degree of heightened variability since 1900 CE and more extreme westward migrations. Consequently, these changes to the NASH have substantial implications for regional hydroclimatic shifts across North America with the potential to increase the exposure and vulnerability of communities and economic sectors to climate risks.

## MATERIALS AND METHODS

### Experimental design: Field methods

For this reconstruction, we gathered 109 chronologies from the International Tree-Ring Data Bank. Given that the NASH is the dominant driver of summer precipitation variability in the southeastern US (*7*, *8*), we targeted species that occur within the domain of the flank of the NASH in the southeastern US (fig. S1). In addition, we sampled multiple species across Georgia, South Carolina, and North Carolina to develop new or update existing chronologies (*n* = 13), giving us a total of 122 chronologies. Using 5.15-mm Haglöf increment borers, we extracted at least two cores per tree, sampling from opposing sides at ~1.5 m above the base (*55*). Of the 122 chronologies, only 33 were retained in the reconstruction model (see the “Statistical analysis: BHI reconstruction” section below; table S1).

### Experimental design: Laboratory methods

We prepared samples for the analysis following standard dendrochronological techniques (*56*). We mounted cores and cross sections for stability while sanding samples with progressively finer sandpaper (to 600 grit as defined by the American National Standards Institute) to reveal the ring structure (*55*–*58*). We visually cross-dated and scanned samples using WinDendro (v. 2017a) to measure the total ring width. Next, we used the program COFECHA (*59*) to statistically confirm the visual cross-dating of the samples, starting with cores from living trees (most recent year: 2018). While many samples were previously cross-dated for earlier studies, we expanded the regional chronology by including new chronologies, thereby increasing our sample depth back to the mid-12th century (fig. S2C). Further, by including updates to previously published chronologies, we were able to expand the calibration model to include data up to 2009. After cross-dating all samples, we detrended each series using ARSTAN (*60*) to fit a “two-thirds” spline (*61*), with the recommended adjustment (*62*), to remove as many biological and nonclimatic growth signals from measurements of total ring width.

### Statistical analysis: Calculating the BHI

Different metrics have been used to characterize the NASH. For example, Li *et al.* (*7*) used the 850-hPa geopotential heights to represent the flank, whereas Diem (*30*) used sea level pressure and 850-hPa geopotential heights to derive the latitude and longitude of the flank. We used the BHI, as it serves as a standardized and integrated representation of variations in east-west flank behavior. While a similar index for the flank exists, albeit at the 850-hPa pressure level and centered over the Southeast US (*30*), that is strongly correlated with the BHI (*r* = 0.77, *P* < 0.05; fig. S8), the latter has been shown to represent the connections between the NASH and different patterns in the climate system, such as temperature (*7*, *25*, *26*), precipitation (*3*–*12*, *25*–*32*), tropical cyclones (*38*, *39*), and streamflow (*41*). It has also been identified as a major predictor of crop yield in the Southeast US (*17*). The BHI is most frequently defined as the zonal pressure gradient between Bermuda and New Orleans, Louisiana, US (*6*, *25*). Given that the center of the summer NASH is positioned approximately 2500 km east of Bermuda (*30*), the BHI can be used to assess east-west changes in the flank of the NASH. This index does not represent the NASH in its entirety. While factors other than the NASH can influence the BHI, such as topography (*7*) and irregular boundaries due to other pressure systems over daily intervals (*27*), the strong field correlations between the BHI and MSLP (figs. S4 and S7) and other flank metrics (e.g., 850-hPa geopotential heights; fig. S4) and prior work (*5*, *6*, *25*, *30*) demonstrate that the index is a good approximation of and a robust metric for assessing east-west behavior in the flank.

Prior work characterizing the relationship between westward migrations and NASH intensity (*14*, *15*) permits us to interpret the behavior of the NASH, albeit broadly, using the BHI. We extracted the mean monthly MSLP (in hPa) for Bermuda (35°N, 65°W) and New Orleans (30°N, 90°W) from the National Oceanic and Atmospheric Administration’s (NOAA’s) National Center for Environmental Prediction/National Center for Atmospheric Research Reanalysis v2 derived surface dataset for 1948–2018 CE (*63*). For each year (*i*) and using a reference period of 1981–2010, we calculated the MJJ (*j*) BHI as follows

$${\text{BHI}}_{i,j}=\frac{{\text{ΔMSLP}}_{i,j}-{\bar{\text{ΔMSLP}}}_{j}}{s_{\text{ΔMSLP},j}}$$ 1

where ΔMSLP is the monthly MSLP between Bermuda and New Orleans, $\bar{\text{ΔMSLP}}$ is the mean difference between Bermuda and New Orleans for the reference period (1981–2010), and *s* is the SD of the MSLP difference between the two sites for the reference period (1981–2010). Given the southerly circulation of the anticyclone, the differences are calculated by subtracting the MSLP in New Orleans (low pressure) from the MSLP in Bermuda (high pressure). The result is the BHI, which is standardized as a *z*-score of the pressure difference. Monthly values can be averaged to get seasonal BHI values (e.g., MJJ). Negative and positive BHI values represent muted and enhanced pressure gradients, respectively. That is, negative values indicate westward migration, while positive values indicate eastward migration.

### Statistical analysis: BHI reconstruction

We reconstructed the average BHI from MJJ, as this period includes the peak in flank intensity, i.e., maximum westward extent during the year (*13*, *25*, *64*). Moreover, this period overlaps with months of increased precipitation across the southeastern US (*65*) and coincides with the period during which changes in the NASH exhibit the greatest influence on precipitation patterns (*5*–*12*, *64*). While other seasonal windows were examined, MJJ yielded the strongest results that were still climatologically important for NASH variability. In addition, although tropical cyclones are an important hydroclimatic feature in the Southeast US, their contributions to mean annual rainfall totals often rapidly diminish from the coast (*38*), and, therefore, the effect of tropical cyclone precipitation is small for the trees used in this study.

To reconstruct the MJJ BHI, we used principal component (PC) regression in the PPR software (*66*–*68*). This method allows us to use many strongly correlated chronologies by combining standardized tree-ring measurements into orthogonal PC scores that are used as predictors of instrumental data over the calibration period. Moreover, it retains only the chronologies that exhibit a strong to moderate correlation with the climate variable, removing unrelated records from the model. Only 33 of the 122 chronologies we collected were retained in the reconstruction model, including 5 updated for this study (table S1). We calibrated our model using BHI values from 1948 to 2009, as this was the common period for the greatest number of chronologies; the latter half of the calibration period (1980–2009) was used for verification (*69*). Given the difference in the most recent year among the chronologies, we used a forward nest approach to reach 2009. As we approach the present, chronologies are removed from the model upon reaching a year that is greater than their most recent year (e.g., a chronology ending in 2000 would not be included in the iteration for 2001). Ultimately, we had 50 nests, 25 of which were forward nests (fig. S2D).

Normally, the coefficient of determination (*R*^2^), reduction of error (RE) (*69*), and coefficient of efficiency (*70*) statistics are used to evaluate the predictive skill of a model. While the RE and coefficient of efficiency values remain positive throughout our reconstruction, given the short common period during the first forward nest (1948–1973 CE), we primarily used the *R*^2^ and the cross-validation calibration reduction of error (CVRE) statistic for predictive skill evaluation, both of which were positive (fig. S2D) (*68*, *71*, *72*). CVRE is calculated by removing one value in the common period during the cross-validation process (*68*, *71*, *72*). In addition, using both the current (*t*) and lagged (*t* + 1) MJJ BHI values produced stronger predictive ability, likely because the NASH modulates multiple environmental variables (*6*–*8*, *25*–*27*, *38*) that control annual wood production (*21*) during the current and following year, allowing for a persistent climate signal. We limit our reconstruction to 1140–2009 CE because the CVRE values are negative due to the limited number of chronologies extending beyond 1140 CE. As the reconstruction is based on the first nest (1948–1973 CE), we report an explained variance of 71% (*r* = 0.84). However, correlating all nests during the instrumental period (1948–2009 CE; fig. S2), we report an explained variance of 62% (*r* = 0.79). Last, we compared the reconstructed and instrumental BHI values using field correlations between the BHI and various climate variables using KNMI Climate Explorer (*73*) to assess whether the spatial patterns and correlation coefficients were similar between the two BHI records.

In addition to using the annual record to explore high-frequency variations and trends, we calculated 10- and 30-year running means for the BHI to understand decadal and multidecadal patterns. To examine anomalies in the flank position, we used Climate Data Toolbox (*74*) for MATLAB. We also calculated trends in the BHI using a least squares approach, which is available in the Climate Data Toolbox (*74*). We also divided the time series into preindustrial (1140–1850 CE) and 20th-century (1900–2009 CE) intervals to determine whether there were trends in these data subsets. Likewise, we examined the presence of linear trends in decadal, multidecadal, and centennial timescales. To examine trends in various portions of the distribution of reconstructed BHI values, we used quantile regression (*75*) in the R package “quantreg” (*76*). Quantile regression has the benefit of providing trend estimates across the probability distribution of the reconstructed BHI values, thus allowing us to examine trends in other portions of the distribution rather than the conditional mean, which is what ordinary least squares regression provides. We estimate the linear relationship between quantiles of the reconstructed BHI values and time. We use quantiles ranging from 0.01 to 0.99, focusing on the lower extremes of the BHI because that indicates when the BHI is in a more westerly location and, thus, more likely to affect the Southeast US and the Caribbean.

Last, in addition to trend analyses, we examined whether the mean and variance of the 20th-century BHI differed from the preindustrial BHI. We used a one-way analysis of variance (ANOVA) to determine whether there were any differences in the preindustrial and 20th-century averages. This was repeated for the MCA and LIA and the averaged time series. To assess whether there was a difference in variability between the 20th century and preindustrial periods, we used a one-tailed two-sample *F* test, as prior work suggests the NASH will continue exhibiting increased variability in response to anthropogenic forcing (*7*, *14*, *15*). We repeated these analyses for the MCA, LIA, and averaged time series. In addition, we used a normal kernel function to further compare the BHI during the 20th century and the preindustrial period. We further divided the preindustrial period into segments spanning 110 years, which is the length of the 20th-century interval, to examine this signal over shorter timescales during the preindustrial period.

### Statistical analysis: Climate connections

Changes to the climate system over different spatiotemporal scales can be attributed to external forcing mechanisms (e.g., solar insolation, volcanic eruptions, and anthropogenic greenhouse gasses). Previous studies have found connections between major eruptions and regional to large-scale climate variability (*22*, *39*, *77*–*80*). To understand the connections between NASH flank variability and external forcings, we performed a Superposed Epoch Analysis (SEA) between major volcanic eruptions and the reconstructed BHI (fig. S6E and table S3). SEAs are often used to examine time-dependent correlations (i.e., lag-lead relationships) between event data (e.g., volcanic eruptions) and sequential data (e.g., reconstructed BHI time series) using a window centered around the event. To run the SEA, we used the “sea” function in the R package “dplR” (*81*). We compiled a list of major tropical volcanic eruptions (table S2) (*44*, *45*) that occurred during our reconstruction period, defining “major eruptions” as those with a Volcanic Explosivity Index (VEI) ≥ 5 (≥1 km^3^ of ejected tephra volume), with VEI 5 eruptions being restricted to Central America. Eruptions of these magnitudes often result in regional to global climate variability (*82*), some of which have been historically documented. In addition, if two or more eruptions occurred within 10 years of one another, then we only included the strongest eruptions, e.g., the 1815 eruption of Tambora (VEI 7) instead of the 1808 mystery eruption (VEI 6) (*83*). Of note is the exclusion of the 1982 eruption of El Chichón. We elected to exclude this eruption despite including the ~1345 CE eruption because the latter produced an eruption column that extended farther into the stratosphere than any during the 1982 sequence based on analysis of tephra deposits (*84*, *85*). We included 14 major eruptions in our SEA (table S2). Given the limited sample size, we used a significance threshold of 0.1; however, we also delineated the boundaries of 0.05 and 0.01 to aid with our analysis.

Given the importance of the NASH flank in driving temperature and precipitation variability in the southeastern US, we examined our reconstruction in the context of records of temperatures and moisture metrics, i.e., PDSI and SPEI. As both PDSI and SPEI incorporate temperature in the form of potential evapotranspiration, we primarily focus on the relationship between BHI and these moisture metrics (Fig. 5 and fig. S12, B and C). While there are numerous annually resolved hydroclimate reconstructions in the Southeast US, most of our chronologies have previously been incorporated into these regional reconstructions, e.g., Living Blended Drought Atlas (*86*). Consequently, the high degree of overlap between our BHI reconstruction and other hydroclimatic records would result in artificially inflated correlation coefficients. To prevent this, we used outputs of mean June to August (JJA) 2-m temperatures, mean JJA PDSI, and mean JJA (3-month timescale) SPEI from the PHYDA product (Fig. 5 and figs. S11 to S16) (*42*). Briefly, PHYDA is built using data assimilation techniques that combine multiple annually resolved proxies (*n* = 2978) with the Community Earth System Model Last Millennium Reanalysis that produces a 2000-year field of hydroclimate (i.e., PDSI and SPEI) and the associated atmosphere-ocean conditions [i.e., ITCZ index, sea surface temperature indices in El Niño–Southern Oscillation regions and the North Atlantic, and surface air temperatures]. For a full description of PHYDA, see Steiger *et al.* (*42*).

As PDSI has a longer memory than 3-month SPEI, we use the latter to capture hydroclimatic changes while reducing the influence of prior conditions (i.e., a wet spring and a dry summer). At each point, we calculated the Pearson’s correlation coefficient between the detrended PHYDA variables and our reconstruction during the period of overlap (1140–2000 CE). Likewise, we calculated the Pearson’s correlation coefficient to determine whether there was any connection between the flank and the latitude of the ITCZ, which was included in the PHYDA outputs (*42*). PHYDA calculates the latitude of the ITCZ using a spatial weighting approach to determine the latitude of the maximum precipitation value across tropical latitudes for each of the 11 longitudinal sectors (*42*, *87*). As previous studies have connected precipitation variability in the region to changes in both the NASH and the ITCZ (*43*), we accounted for changes in the ITCZ position using a Pearson’s partial correlation. In addition to looking at relationships over annual intervals, we also calculated the 5-year mean for each time series to identify intradecadal patterns over the past 870 years, calculating correlation coefficients between each variable and our reconstruction (fig. S12).

To understand changes in temperature, PDSI, and SPEI in response to volcanically driven shifts in the flank, we calculated the anomalies in the three climate variables in the 5 years before (*t*_0_ – 5 to *t*_0_ – 1), the year concurrent with (*t*_0_), and 5 years following (*t*_0_ + 1 to *t*_0_ + 5) an eruption (Fig. 6 and figs. S17 to S23). These anomalies were based on the detrended means for the longest overlapping period with our reconstruction, 1140–2000 CE. In addition, we calculated the mean for the 5 years before an eruption (*t*_0_), which was also used to calculate the relative anomalies for conditions at *t*_0_ – *t*_0_ + 5. This approach has the benefit of looking at changes following specific eruptions and was modified after (*45*). Lastly, following the methods outlined by Tejedor *et al.* (*45*), we calculated composite anomalies averaged across all eruption periods (i.e., for all 14 eruptions, including lags shown in Fig. 6E). This approach allowed us to examine the average BHI change experienced in the posteruption period and during the individual lags (e.g., the average hydroclimatic change at lag + 1). Similar to the individual anomalies discussed above, we calculated the anomalies that were relative to the average across the study period and preeruption years.

## Supplementary Materials

### The PDF file includes:

Supplementary Text Figs. S1 to S23 Table S1 to S3 Legend for References S1

### Other Supplementary Material for this manuscript includes the following:

References S1
