# Supplementary material for: Changes in the western flank of the North Atlantic subtropical high since 1140 CE: Extremes, drivers, and hydroclimatic patterns
Source: Sci Adv. 2025 Apr 16;11(16):eadr5065. doi: 10.1126/sciadv.adr5065 (PMC12002119; doi:10.1126/sciadv.adr5065)
Supplement: Supplementary file 1 — Supplementary Text Figs. S1 to S23 Table S1 to S3 Legend for References S1 [file sciadv.adr5065_sm.pdf]

Science Advances
AAAS

Supplementary Materials for

Changes in the western flank of the North Atlantic subtropical high since 1140 CE: Extremes, drivers, and hydroclimatic patterns

Joshua C. Bregy *et al.*

Corresponding author: Joshua C. Bregy, jbregy@clemson.edu

Sci. Adv. **11**, eadr5065 (2025)
DOI: 10.1126/sciadv.adr5065

The PDF file includes:

Supplementary Text
Figs. S1 to S23
Table S1 to S3
Legend for References S1

Other Supplementary Material for this manuscript includes the following:

References S1

Supplementary Text

Role of the Western Flank: Temperature

Strong to moderate inverse relationships ($-0.5 < r \leq -0.2$; $p < 0.05$) between the reconstructed BHI and summer air temperatures occur throughout the southeastern US over annual and decadal timescales (Figs. S10, S11a). This inverse relationship is also present across the entire eastern US, albeit at a weaker magnitude ($-0.3 < r \leq -0.1$; $p < 0.05$). Likewise, a large area of weak to moderate inverse correlations exists across much of the western and central North Atlantic Ocean. These inverse relationships indicate that air temperature increases in response to the western flank migrating west (i.e., negative BHI). Northwestward positioning of the flank can lead to warmer temperatures as subsidence and reduced large-scale moisture advection increases incoming radiation by diminishing cloud cover (6, 10, 15). Conversely, an eastward positioning of the flank directs moisture into the eastern US, increasing cloud cover and reducing temperatures. Likewise, depending on the extent of its eastward position, circulation associated with the NASH could provide support for thermal advection transporting heat away from the region, especially if surface cyclonic circulation is positioned farther west.

Elsewhere across the basin, air temperatures tend to increase and decrease as the flank migrates east (positive BHI) and west (negative BHI), respectively. This relationship is strongest in the western Gulf of Mexico, across most of eastern Mexico, and portions of New England and Eastern Canada ($0.1 \leq r \leq 0.3$; $p < 0.05$; Figs. S10, S11a). The positive correlations between the BHI and air temperature reflect increased moisture transport to regions located on the periphery of the western flank (6–8) and the advection of heat away from the region. Conversely, when the flank is positioned farther east, its influence on temperature in the region weakens.

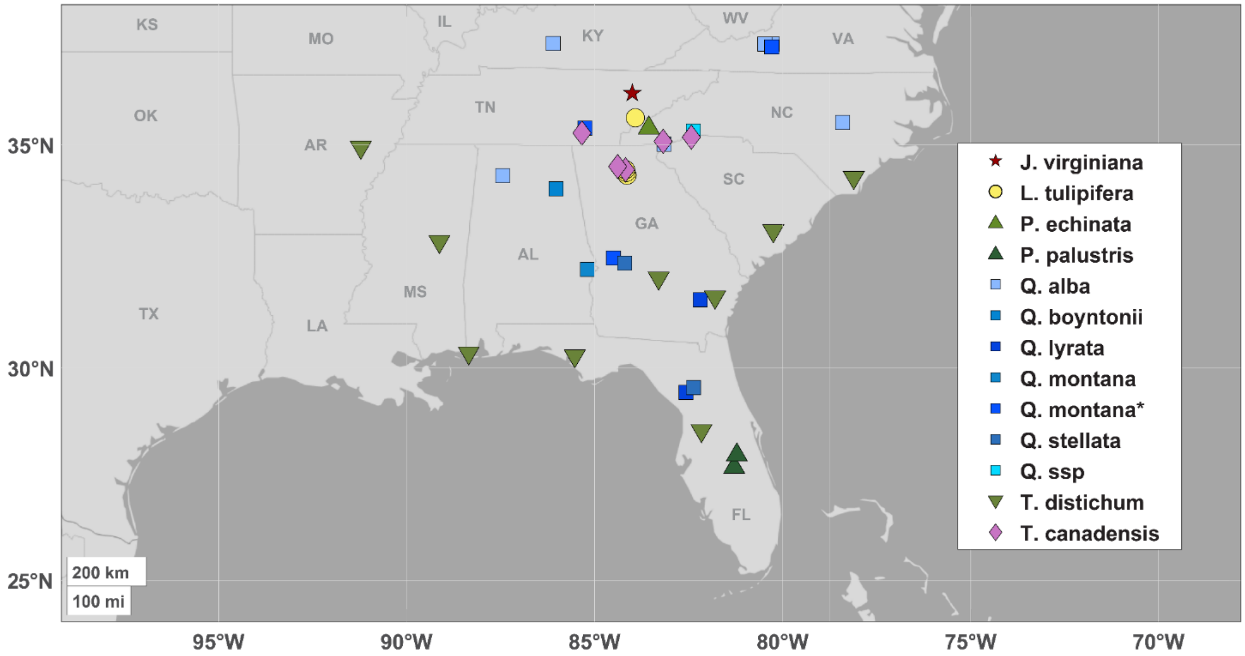

Fig. S1.

Chronology locations. Map showing the location and species for the 33 chronologies included in the reconstruction model. Starred (*) entries for *Quercus montana* indicate sites with *Quercus prinus* listed as the species. For a list of the incorporated chronologies used, including the source, refer to Table S1.

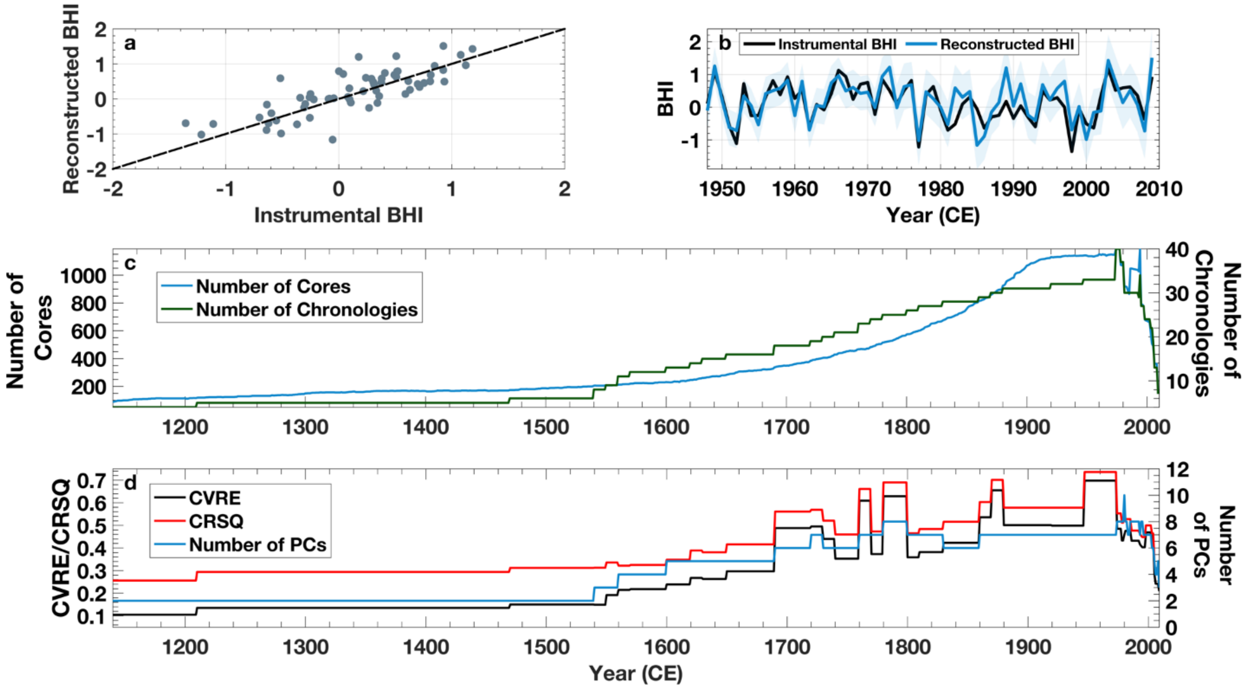

Fig. S2.

Reconstruction statistics. Comparison between the instrumental and reconstructed BHI with associated reconstruction statistics. **(a)** Scatter plot between instrumental BHI and reconstructed BHI values from 1948 to 2009 CE (r = 0.79; p << 0.01; mean absolute error = 0.072). The explained variance for all the nests during the instrumental period is 62 %, while the explained variance for the first nest, which is used to reconstruct pre-instrumental BHI, is 71 % (r = 0.84; see text). Dashed line is the 1-to-1 line. **(b)** Time series plot of the instrumental BHI (black line) and reconstructed BHI (blue line) from 1948 to 2009 CE. Blue shading represents the confidence intervals for reconstructed BHI. **(c)** Plot of the number of core samples (blue line; left y-axis) and the number of chronologies (green line; right y-axis) during the entire study period (1140–2009 CE). **(d)** CVRE (black line; left axis), CR^2 (red line; left axis), and the number of PCs used in each nest (blue line; right axis) for the reconstruction period (1140–2009 CE).

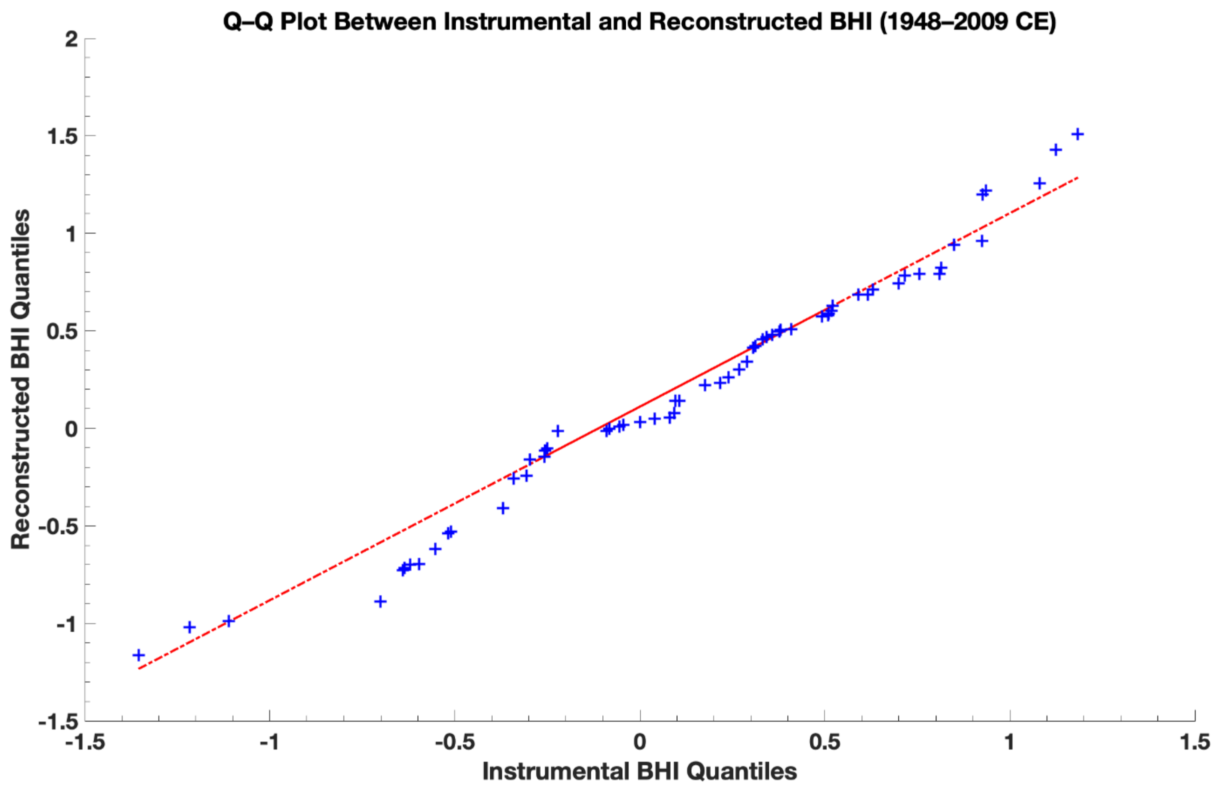

Fig. S3.

BHI QQ plot. Quantile–quantile plot between instrumental and reconstructed BHI (1948–2009 CE).

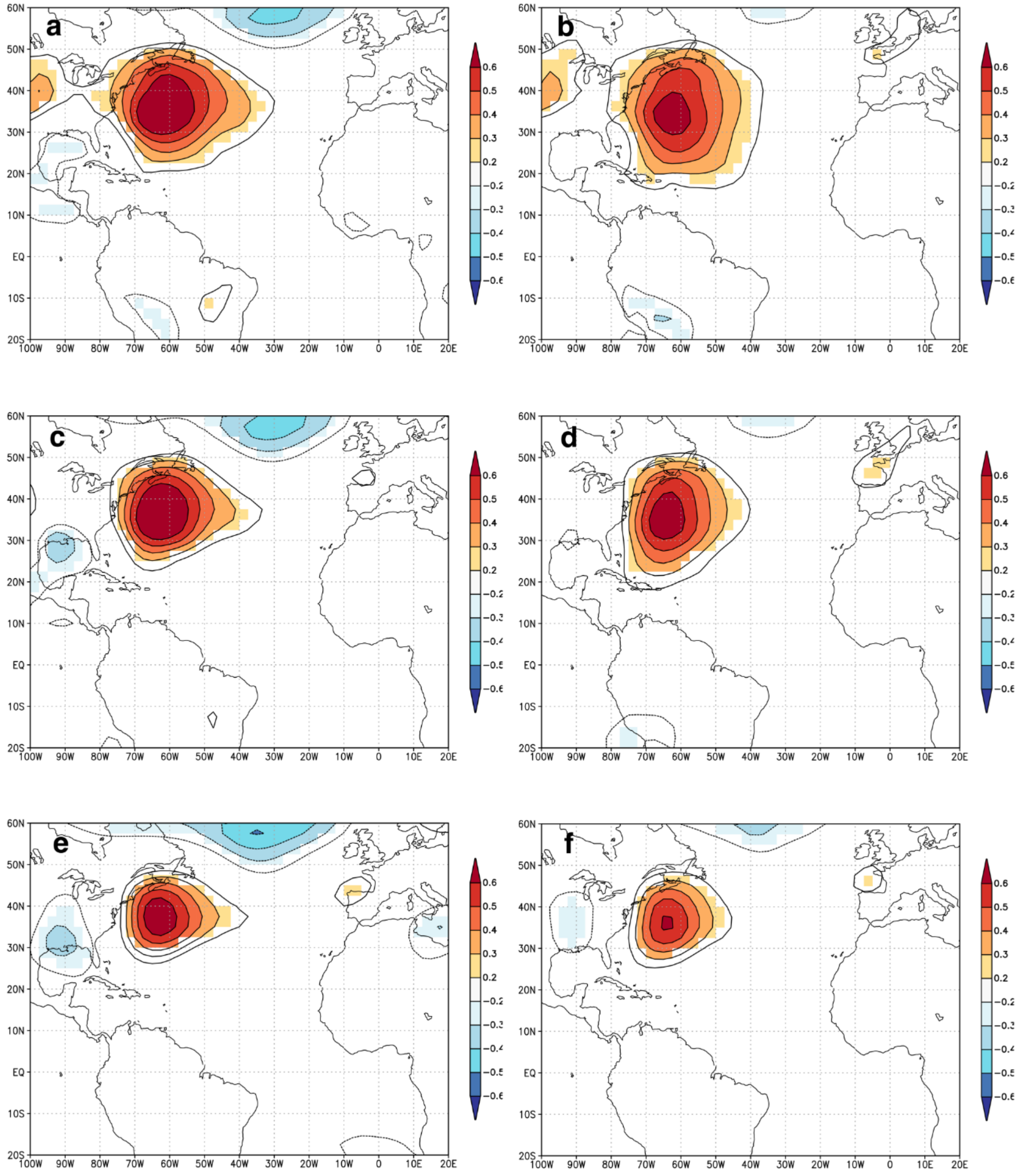

Fig. S4.

Field correlations between the BHI and SLP and geopotential heights. Field correlations between instrumental (**a**, **c**, **e**) and reconstructed (**b**, **d**, **f**) BHI and sea-level pressure and geopotential heights averaged over May–July (1948–2009 CE). **(a)** Correlation between instrumental BHI and sea-level pressure from NCEP NCAR Reanalysis dataset. Only areas that exhibit significant correlations ($p < 0.1$) are contoured. **(b)** Same as in **a** but using the reconstructed BHI. **(c)** Correlation between instrumental BHI and 850-mbar geopotential heights from the NCEP NCAR Reanalysis dataset. Only areas that exhibit significant correlations ($p < 0.1$) are contoured. **(d)** Same as in **c** but using reconstructed BHI. **(e)** Correlation between instrumental BHI and 500-mbar geopotential

heights from the NCEP NCAR Reanalysis dataset. Only areas that exhibit significant correlations ($p < 0.1$) are contoured. **(f)** Same in **e** but using reconstructed BHI. All analyses and figures were completed using KNMI Climate Explorer (73).

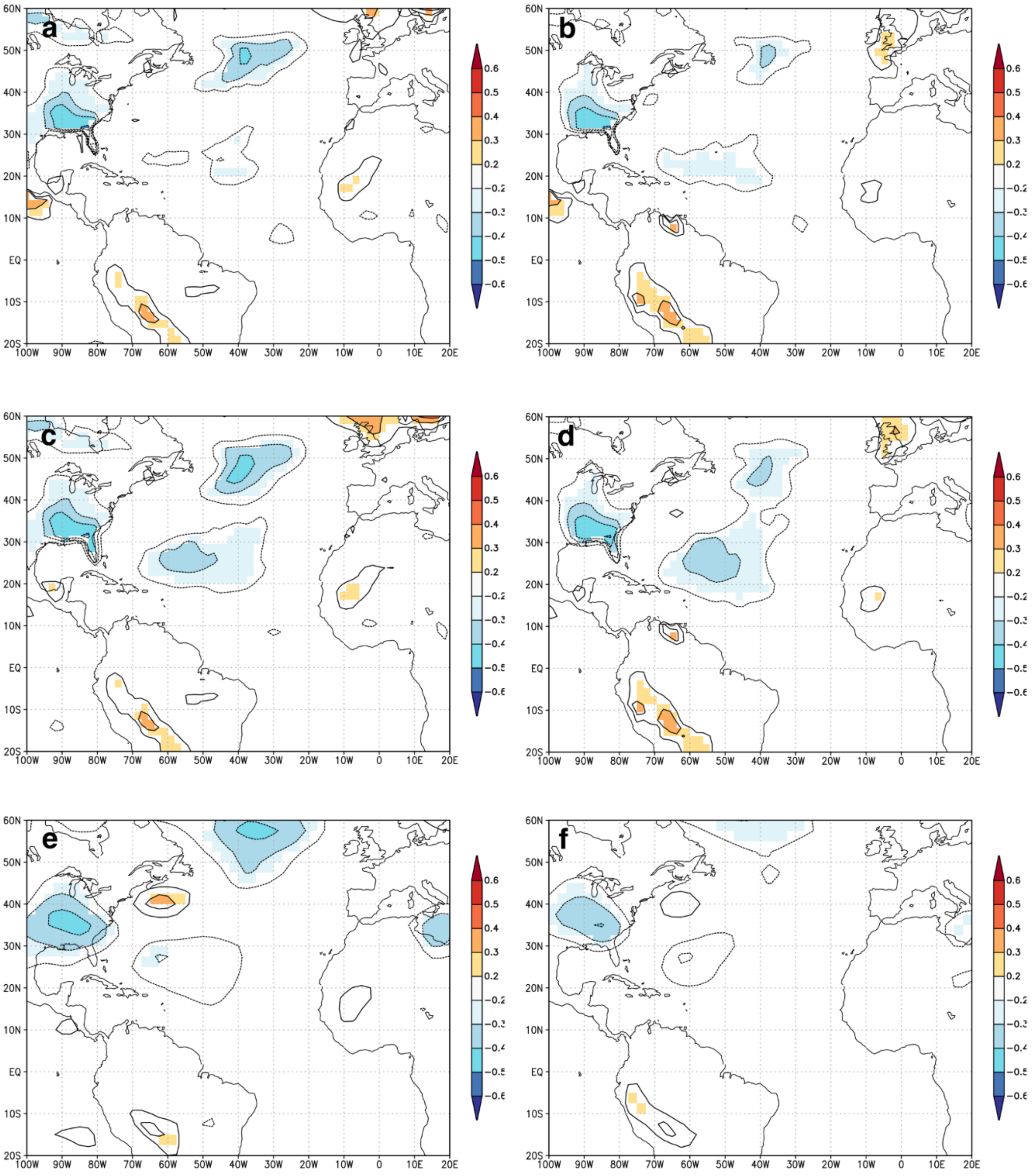

Fig. S5.

Field correlations between BHI and temperature. Field correlations between instrumental (**a**, **c**, **e**) and reconstructed (**b**, **d**, **f**) BHI and temperatures averaged over May–July (1948–2009 CE). **(a)** Correlations between instrumental BHI and surface temperatures (°C) from the NCEP NCAR Reanalysis dataset. Only areas that exhibit significant correlations ($p < 0.1$) are contoured. **(b)** Same as in **a** but using the reconstructed BHI. **(c)** Correlations between instrumental BHI and 2-m temperatures (°C) from the NCEP NCAR Reanalysis dataset. Only areas that exhibit significant correlations ($p < 0.1$) are contoured. **(d)** Same as in **c** but using reconstructed BHI. **(e)** Correlations between instrumental BHI and 850-mbar temperatures (°C) from the NCEP NCAR Reanalysis

dataset. Only areas that exhibit significant correlations ($p < 0.1$) are contoured. **(f)** Same as in **e** but using reconstructed BHI. All analyses and figures were completed using KNMI Climate Explorer (73).

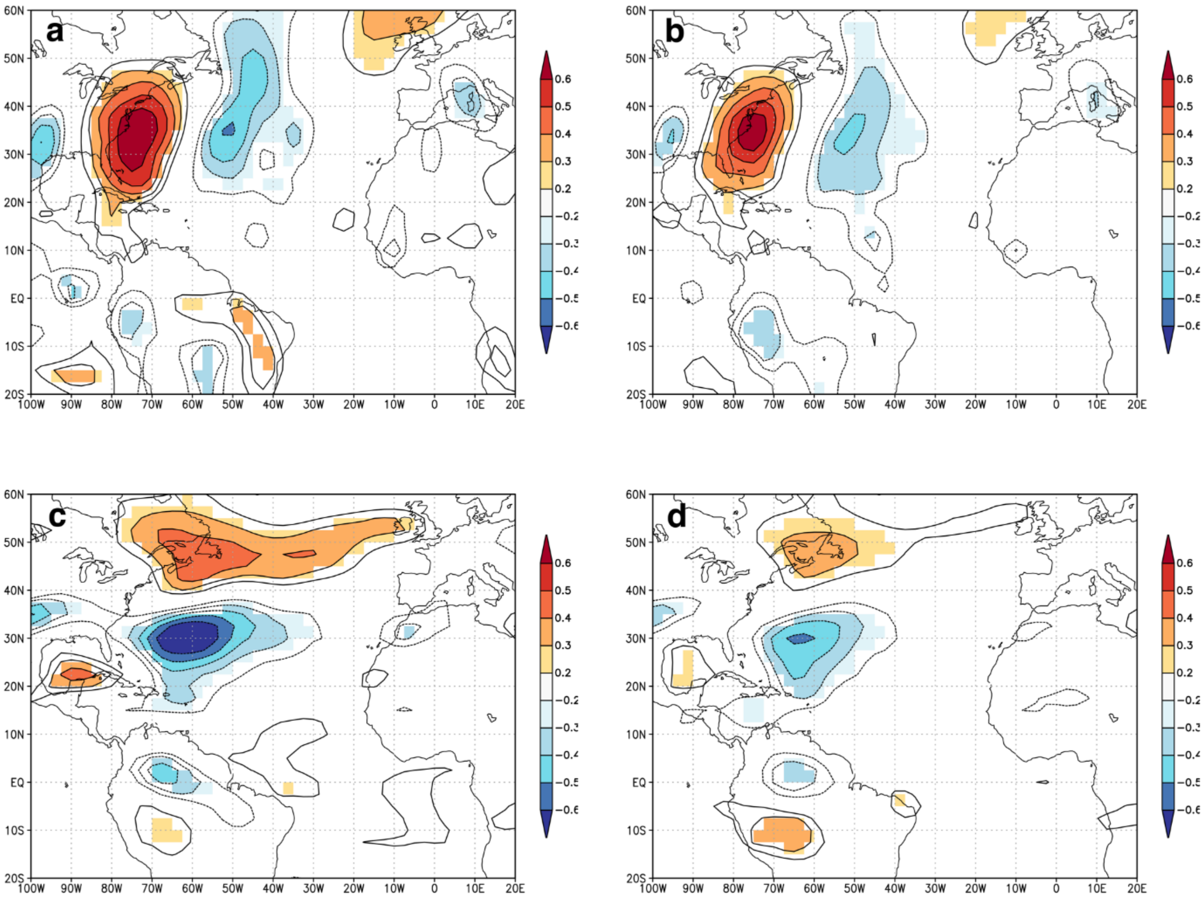

Fig. S6.

Field correlations between BHI and 850-mbar winds. Field correlations between instrumental (**a**, **c**) and reconstructed (**b**, **d**) BHI and 850-mbar meridional and zonal winds averaged over May–July (1948–2009 CE). **(a)** Correlations between instrumental BHI and 850-mbar meridional winds from the NCEP NCAR Reanalysis dataset. Only areas that exhibit significant correlations ($p < 0.1$) are contoured. **(b)** Same as in **a** but using the reconstructed BHI. **(c)** Correlations between instrumental BHI and 850-mbar zonal winds from the NCEP NCAR Reanalysis dataset. Only areas that exhibit significant correlations ($p < 0.1$) are contoured. **(d)** Same as in **c** but using reconstructed BHI. All analyses and figures were completed using KNMI Climate Explorer (73).

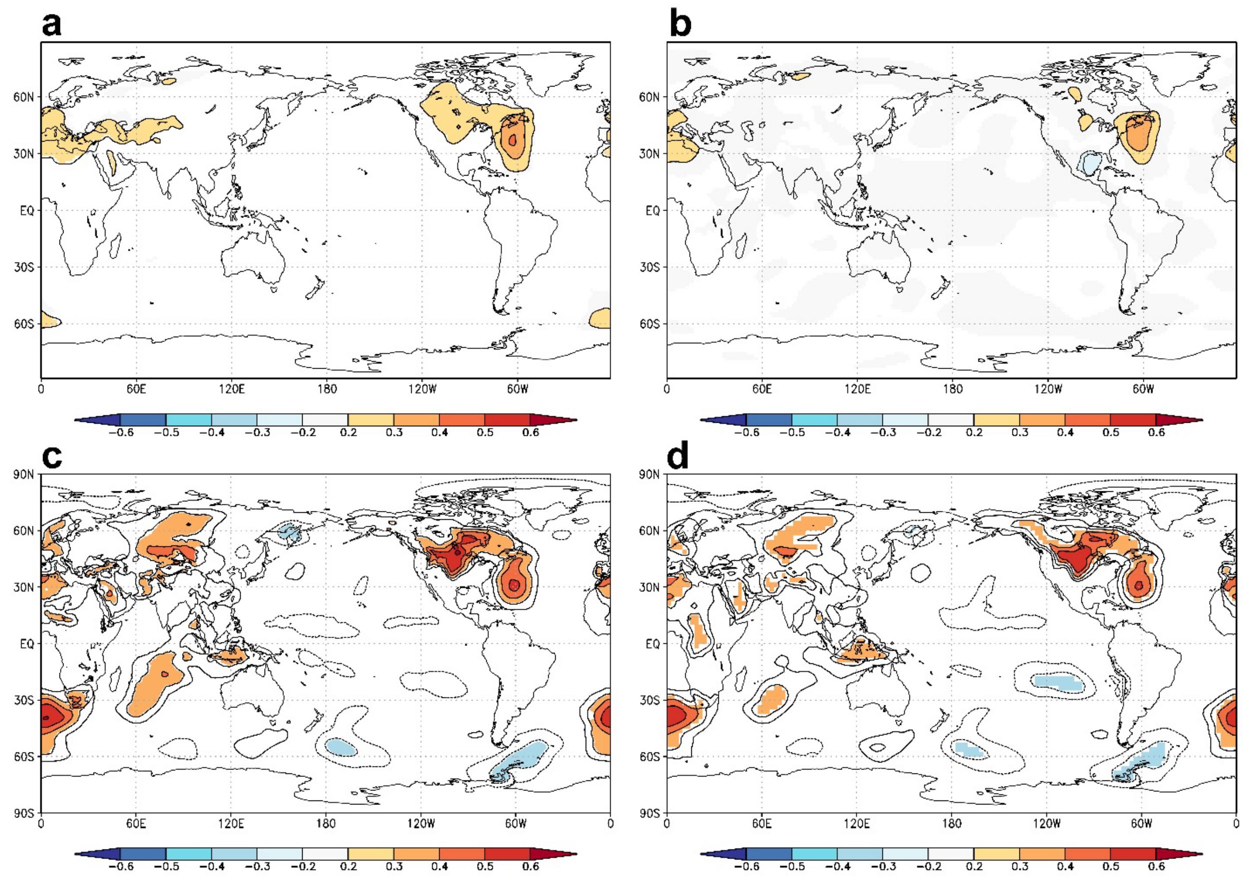

Fig. S7.

Field correlations between reconstructed BHI and mean sea-level pressure (MSLP). Different reanalysis products were used to obtain gridded MSLP values, which were averaged over May–July. **(a)** Correlations between reconstructed BHI and the ERA-20C reanalysis dataset (1900–2009 CE). Only areas that exhibit significant correlations ($p < 0.1$) are contoured. **(b)** Correlations between reconstructed BHI and the CERA-20C reanalysis dataset (1901–2009 CE). Only areas that exhibit significant correlations ($p < 0.1$) are contoured. **(c)** Correlations between reconstructed BHI and the NASA MERRA-2 reanalysis dataset (1980–2009 CE). Only areas that exhibit significant correlations ($p < 0.1$) are contoured. **(d)** Correlations between reconstructed BHI and the NCEP CSFR reanalysis dataset (1979–2009 CE). Only areas that exhibit significant correlations ($p < 0.1$) are contoured. All analyses and figures were completed and made using KNMI Climate Explorer (73).

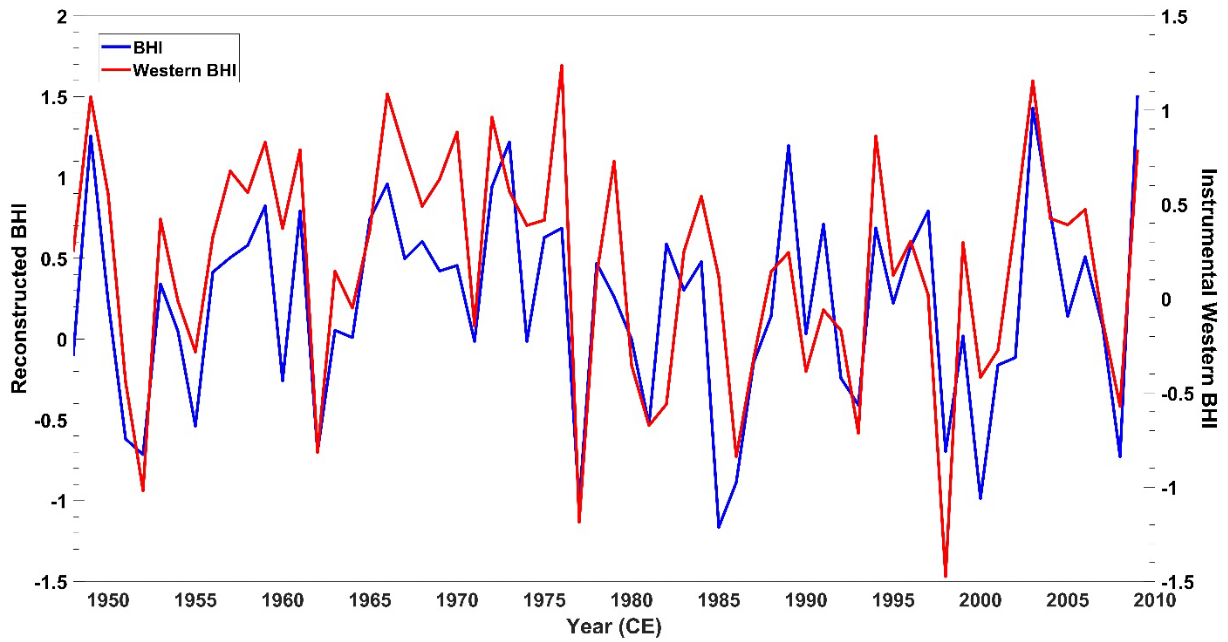

Fig. S8. Time series of the reconstructed BHI and the instrumental Western BHI from 1948 to 2009 CE. The Western BHI is calculated using monthly mean monthly 850-hPa geopotential heights from the NCEP/DOE 1 reanalysis dataset following the methods outlined in reference 30. Values represent MJJ averages.

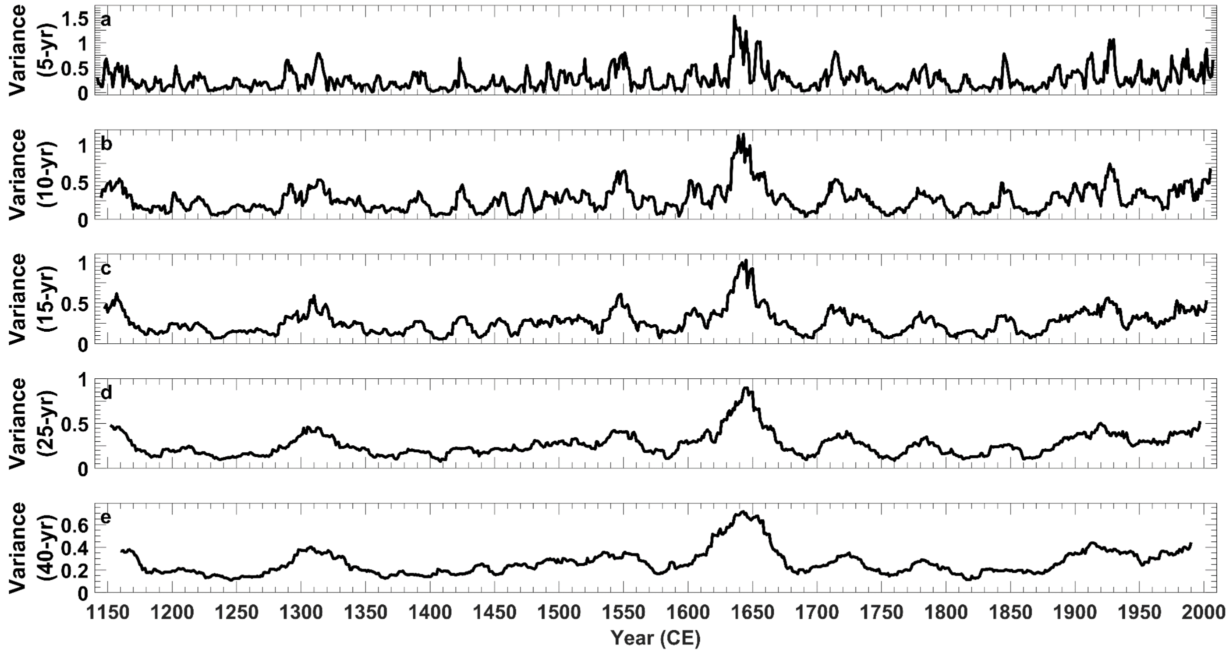

Fig. S9.

BHI moving variance. Time series of BHI variability using different centered window sizes. **(a)** Five-year BHI variance (1140–2009 CE). **(b)** Ten-year BHI variance (1140–2009 CE). **(c)** Fifteen-year BHI variance (1140–2009 CE). **(d)** Twenty-five-year BHI variance (1140–2009 CE). **(e)** Forty-year BHI variance (1140–2009 CE).

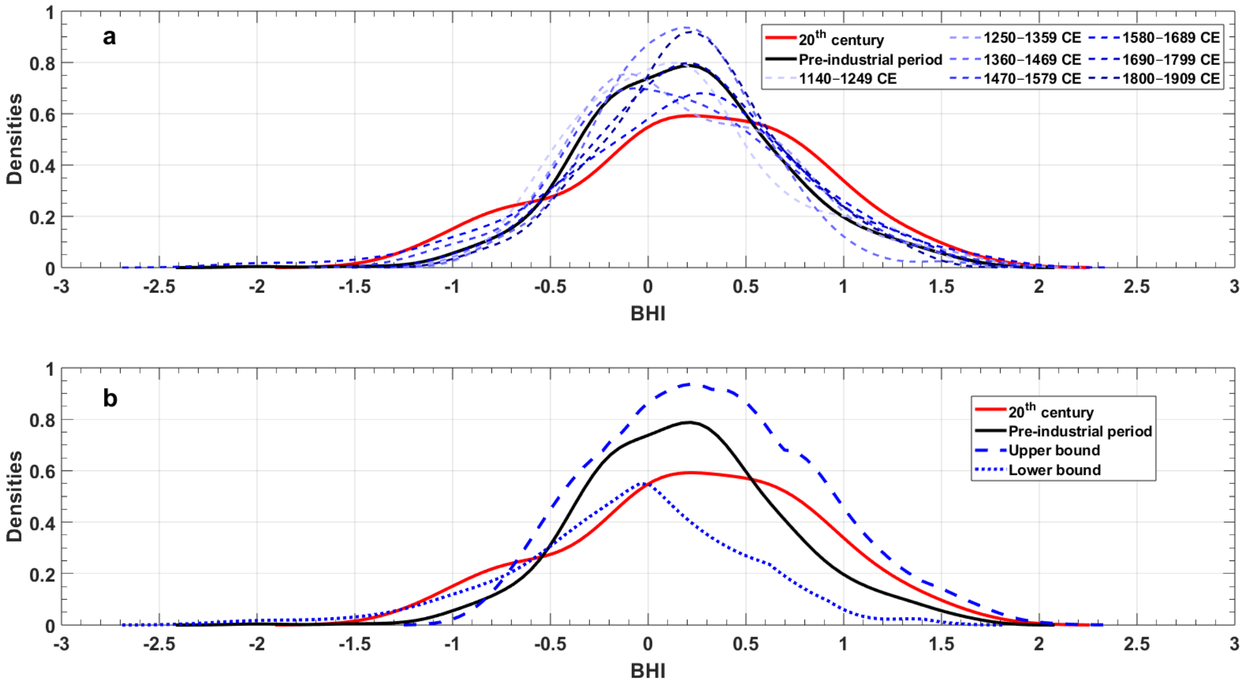

Fig. S10.

Kernal density estimates for the study period. Comparison of kernel density estimations of the BHI during the 20th century (1900–2009 CE; red) and the pre-industrial period (1140–1850 CE; black and blue). **(a)** Kernel density estimates for pre-industrial intervals (blue) grouped by the length of the 20th century estimate (red; 110 years). The entire pre-industrial period is also shown for comparison (black). **(b)** Upper (blue, dashed) and lower (blue, dotted) bounds of the pre-industrial intervals shown in panel **a**.

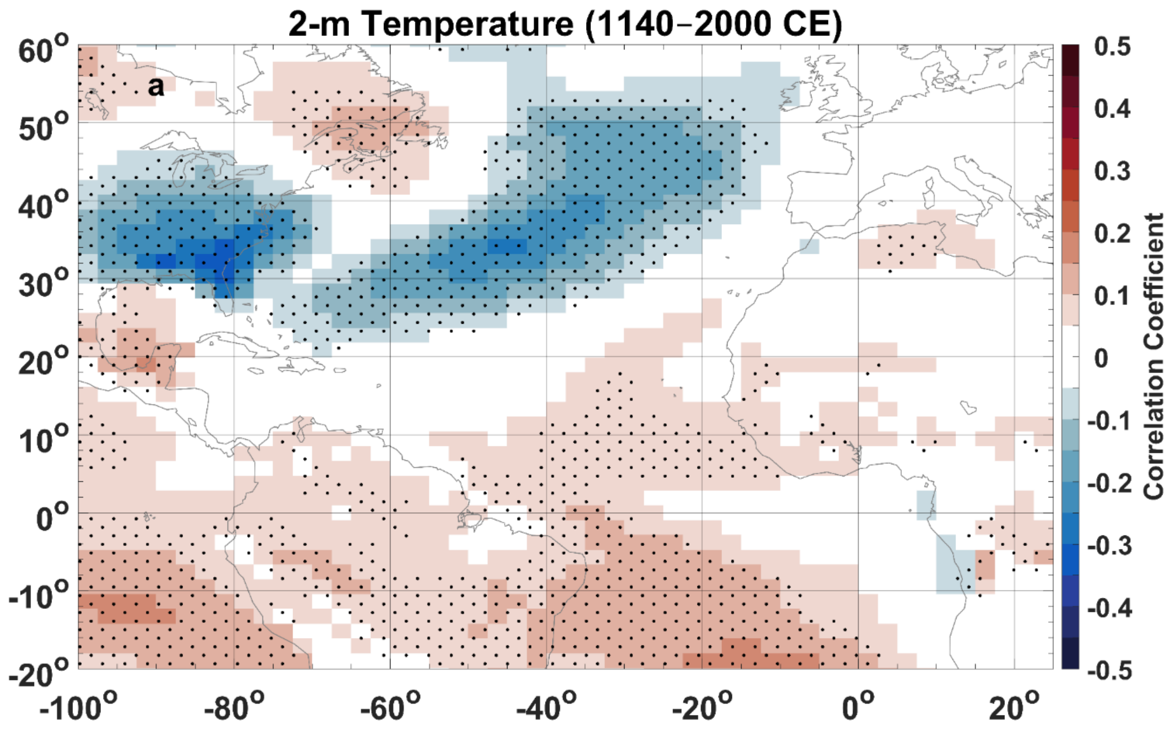

Fig. S11.

Correlations between BHI and 2-m temperature. Field correlation maps between the reconstructed BHI and PHYDA 2-m temperature (°C) outputs from 1140 to 2000 CE. Stippled regions indicate significant correlations ($p \leq 0.05$). The black box indicates the region where the chronologies are located.

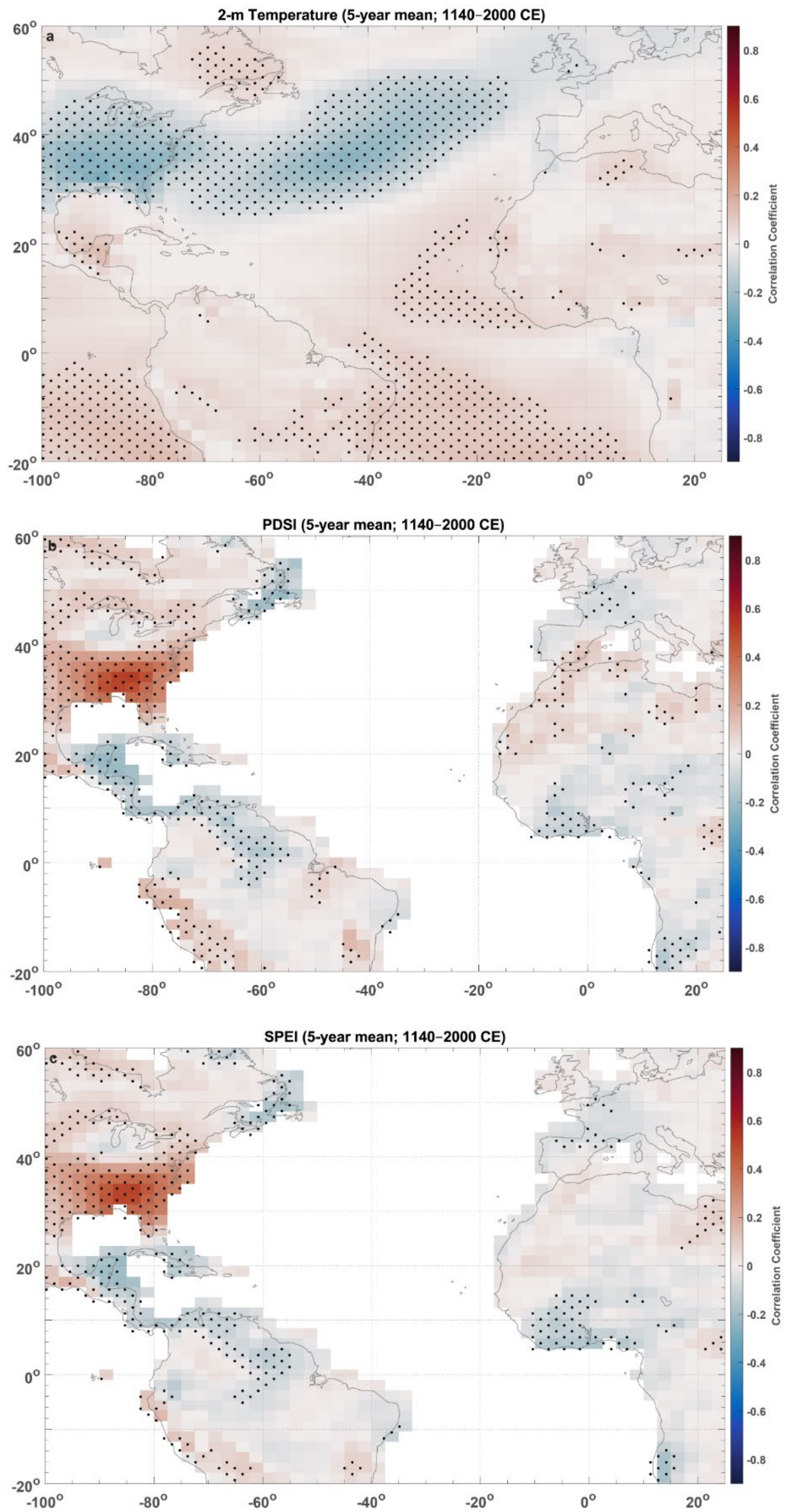

Fig. S12.

Correlations between BHI and PHYDA model outputs (5-year average). Field correlations between the reconstructed BHI and the 5-year averages of PHYDA model outputs from 1140 to 2000 CE. **(a)** Correlations between reconstructed BHI and June–August average 2-m temperatures

(°C). Stippling represents significant correlations ($p \leq 0.05$). **(b)** Correlations between reconstructed BHI and June–August average PDSI. Stippling represents significant correlations ($p \leq 0.05$). **(c)** Correlations between reconstructed BHI and June–August average SPEI. Stippling represents significant correlations ($p \leq 0.05$).

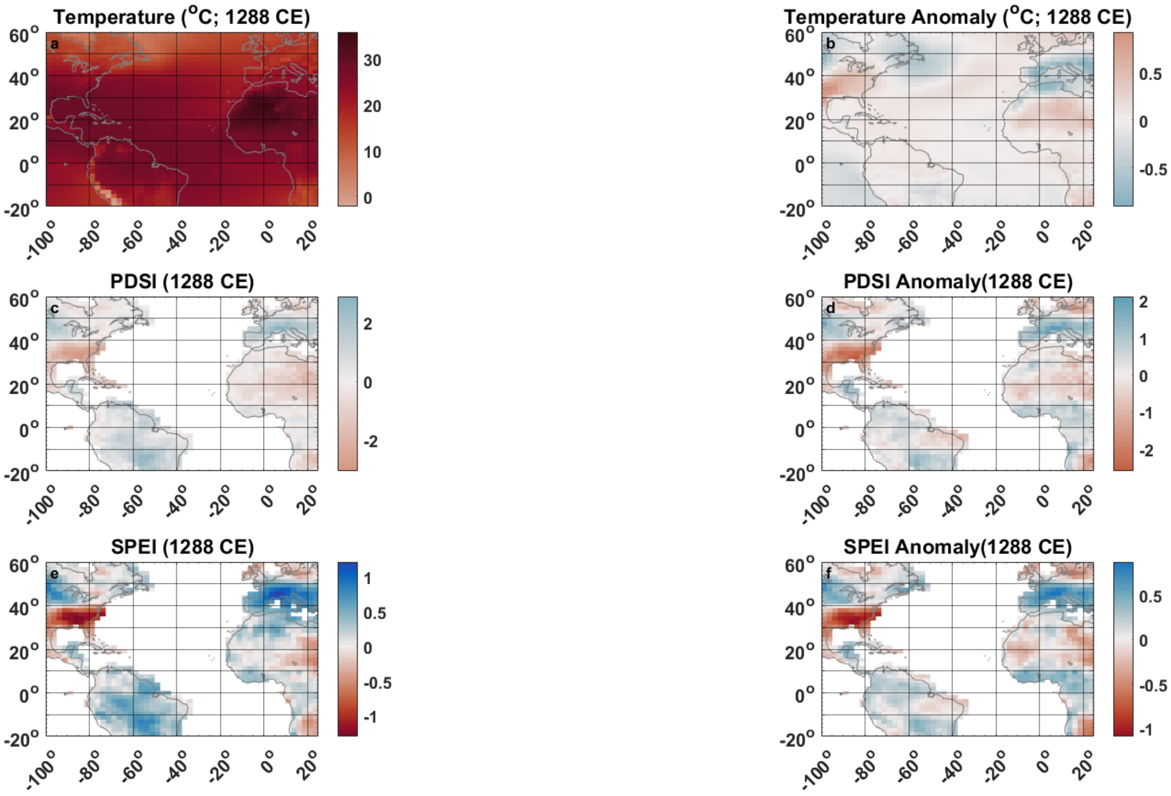

Fig. S13.

Maps of conditions and anomalies in PHYDA outputs in 1288 CE (BHI = -0.7060). (a) Mean JJA 2-m temperatures (°C) during a westward excursion of the western flank. **(b)** Mean JJA 2-m temperature anomalies (°C) during a westward excursion of the western flank. Anomalies were calculated relative to the gridded mean 2-m temperature from PHYDA during the study period (1140–2000 CE). **(c)** Mean JJA PDSI during a westward excursion of the western flank. **(d)** Mean JJA PDSI anomalies during a westward excursion of the western flank. Anomalies were calculated relative to the gridded mean PDSI from PHYDA during the study period (1140–2000 CE). **(e)** Mean JJA SPEI during a westward excursion of the western flank. **(f)** Mean JJA SPEI anomalies during a westward excursion of the western flank. Anomalies were calculated relative to the gridded mean SPEI from PHYDA during the study period (1140–2000 CE).

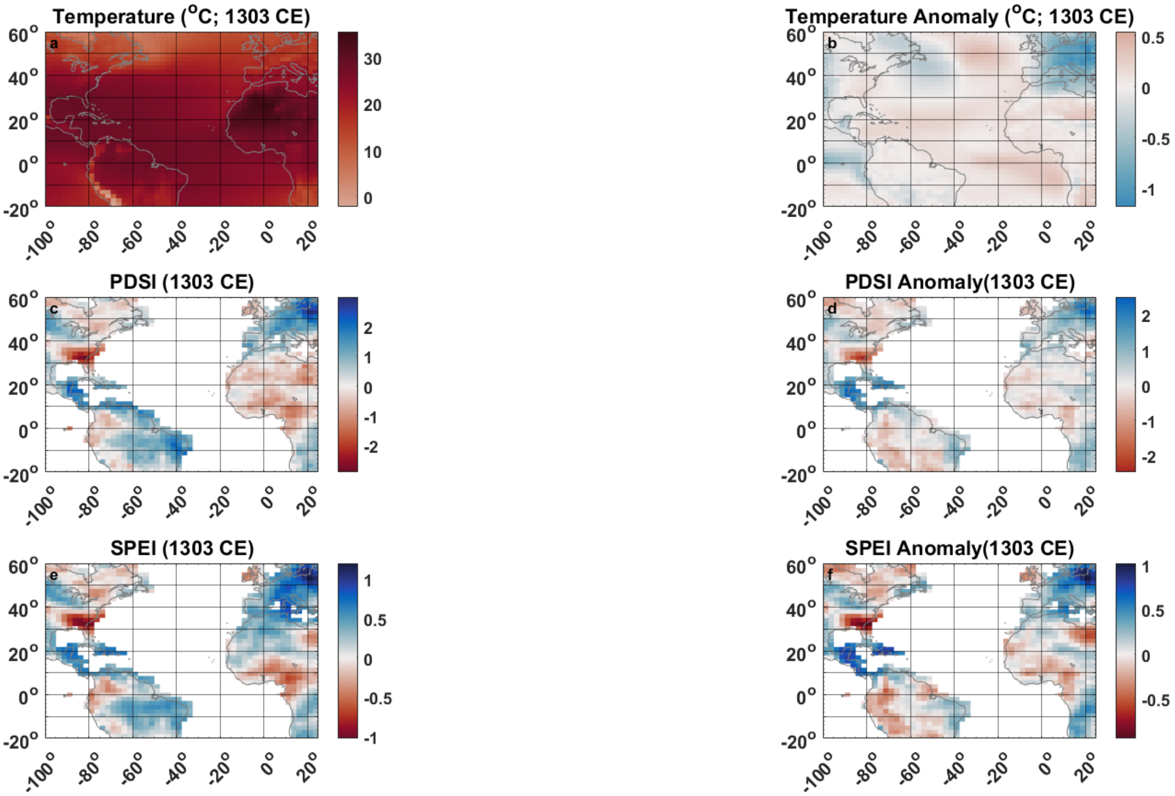

Fig. S14.

Maps of conditions and anomalies in PHYDA outputs in 1303 CE (BHI = -0.7930). (a) Mean JJA 2-m temperatures (°C) during a westward excursion of the western flank. **(b)** Mean JJA 2-m temperature anomalies (°C) during a westward excursion of the western flank. Anomalies were calculated relative to the gridded mean 2-m temperature from PHYDA during the study period (1140–2000 CE). **(c)** Mean JJA PDSI during a westward excursion of the western flank. **(d)** Mean JJA PDSI anomalies during a westward excursion of the western flank. Anomalies were calculated relative to the gridded mean PDSI from PHYDA during the study period (1140–2000 CE). **(e)** Mean JJA SPEI during a westward excursion of the western flank. **(f)** Mean JJA SPEI anomalies during a westward excursion of the western flank. Anomalies were calculated relative to the gridded mean SPEI from PHYDA during the study period (1140–2000 CE).

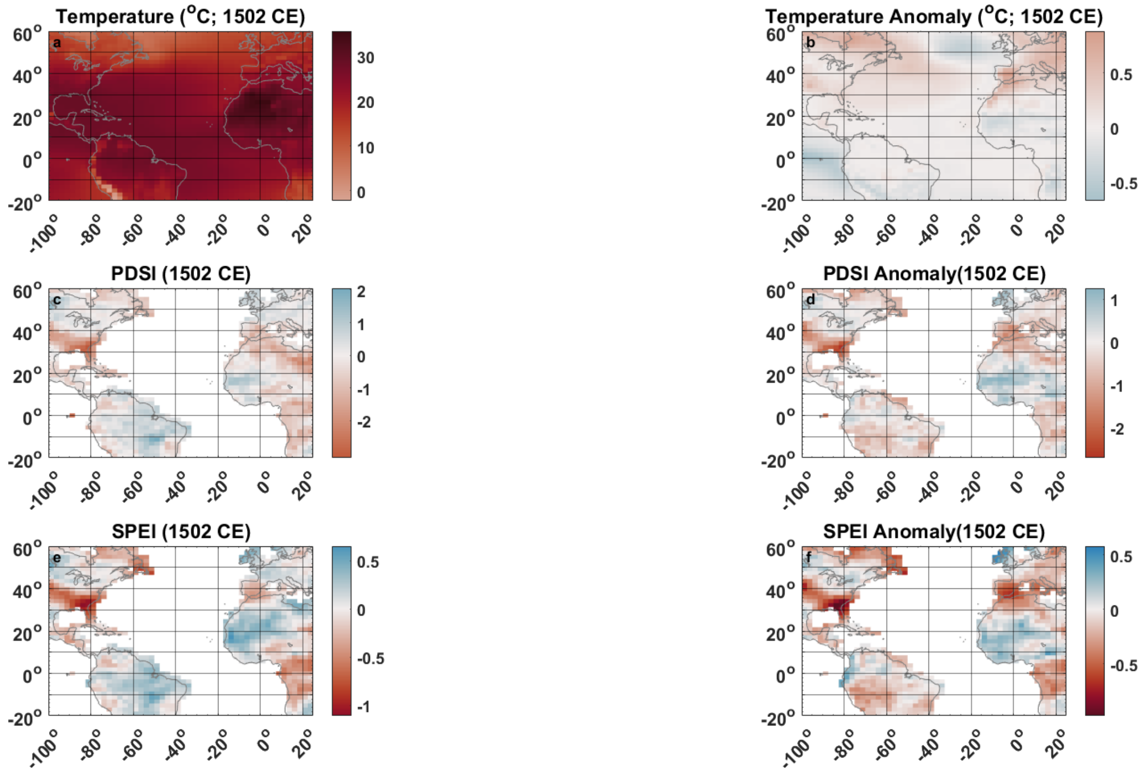

Fig. S15.

Maps of conditions and anomalies in PHYDA outputs in 1502 CE (BHI = -1.0220). (a) Mean JJA 2-m temperatures (°C) during a westward excursion of the western flank. **(b)** Mean JJA 2-m temperature anomalies (°C) during a westward excursion of the western flank. Anomalies were calculated relative to the gridded mean 2-m temperature from PHYDA during the study period (1140–2000 CE). **(c)** Mean JJA PDSI during a westward excursion of the western flank. **(d)** Mean JJA PDSI anomalies during a westward excursion of the western flank. Anomalies were calculated relative to the gridded mean PDSI from PHYDA during the study period (1140–2000 CE). **(e)** Mean JJA SPEI during a westward excursion of the western flank. **(f)** Mean JJA SPEI anomalies during a westward excursion of the western flank. Anomalies were calculated relative to the gridded mean SPEI from PHYDA during the study period (1140–2000 CE).

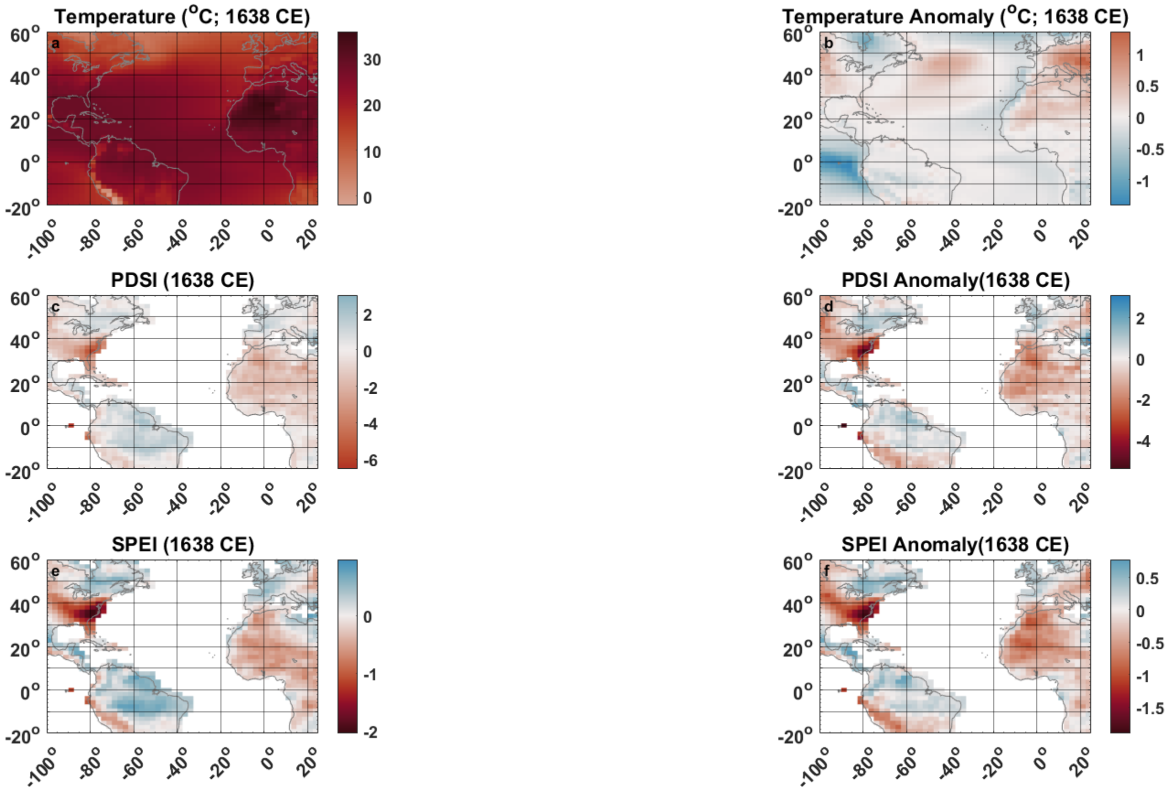

Fig. S16.
Maps of conditions and anomalies in PHYDA outputs in 1638 CE (BHI = -1.9940). (a) Mean JJA 2-m temperatures (°C) during a westward excursion of the western flank. **(b)** Mean JJA 2-m temperature anomalies (°C) during a westward excursion of the western flank. Anomalies were calculated relative to the gridded mean 2-m temperature from PHYDA during the study period (1140–2000 CE). **(c)** Mean JJA PDSI during a westward excursion of the western flank. **(d)** Mean JJA PDSI anomalies during a westward excursion of the western flank. Anomalies were calculated relative to the gridded mean PDSI from PHYDA during the study period (1140–2000 CE). **(e)** Mean JJA SPEI during a westward excursion of the western flank. **(f)** Mean JJA SPEI anomalies during a westward excursion of the western flank. Anomalies were calculated relative to the gridded mean SPEI from PHYDA during the study period (1140–2000 CE).

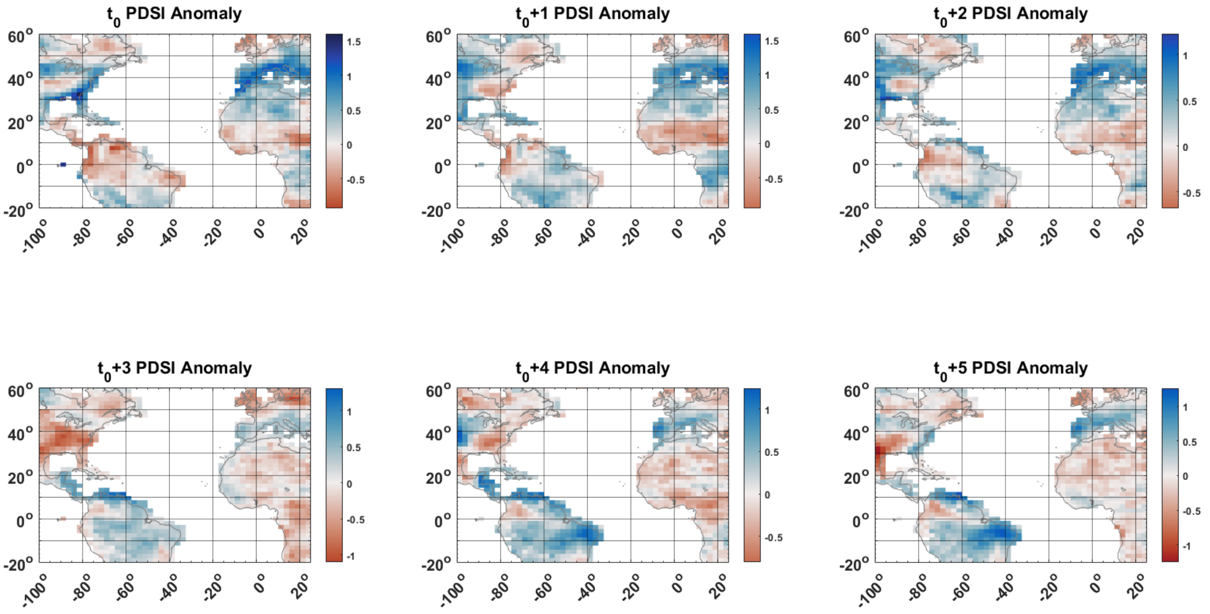

Fig. S17.

Composite PDSI anomalies during and up to five years after an eruption across the study period (1140–2000 CE). Each year in an eruption sequence was grouped by lag year (see Fig. 6e) and averaged before calculating the temperature anomalies. PDSI values are from PHYDA, and anomalies were calculated relative to the gridded mean PDSI of the study period (1140–2000 CE). **(a)** Average PDSI anomaly during an eruption year (t_0). At t_0, the western flank is positioned toward the east (increasing BHI). **(b)** Average PDSI anomaly one year following an eruption (t_0+1), during which the western flank is positioned toward the west (decreasing BHI). **(c)** Average PDSI anomaly two years following an eruption (t_0+2), during which the western flank is positioned toward the west (decreasing BHI). **(d)** Average PDSI anomaly three years following an eruption (t_0+3), during which the western flank is positioned toward the west (decreasing BHI). **(e)** Average PDSI anomaly four years following an eruption (t_0+4), during which the western flank is positioned toward the west (decreasing BHI). **(f)** Average PDSI anomaly five years following an eruption (t_0+5), during which the western flank is positioned toward the east (increasing BHI).

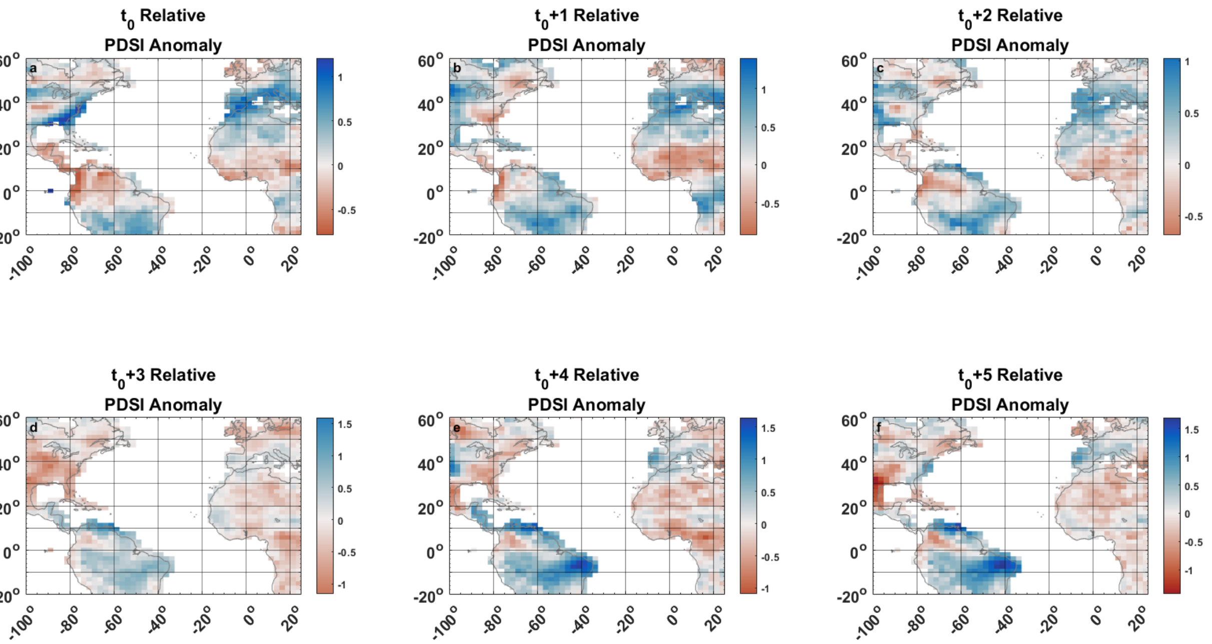

Fig. S18.

Composite relative PDSI anomalies during and up to five years after an eruption across the study period (1140–2000 CE). Each year in an eruption sequence was grouped by lag year (see Fig. 6e) and averaged before calculating the PDSI anomalies. PDSI values are from PHYDA, and anomalies were calculated relative to the gridded mean PDSI of the composite pre-eruption conditions (t_0-5 to t_0-1). **(a)** Average PDSI anomaly during an eruption year (t_0). At t_0, the western flank is positioned toward the east (increasing BHI). **(b)** Average PDSI anomaly one year following an eruption (t_0+1), during which the western flank is positioned toward the west (decreasing BHI). **(c)** Average PDSI anomaly two years following an eruption (t_0+2), during which the western flank is positioned toward the west (decreasing BHI). **(d)** Average PDSI anomaly three years following an eruption (t_0+3), during which the western flank is positioned toward the west (decreasing BHI). **(e)** Average PDSI anomaly four years following an eruption (t_0+4), during which the western flank is positioned toward the west (decreasing BHI). **(f)** Average PDSI anomaly five years following an eruption (t_0+5), during which the western flank is positioned toward the east (increasing BHI).

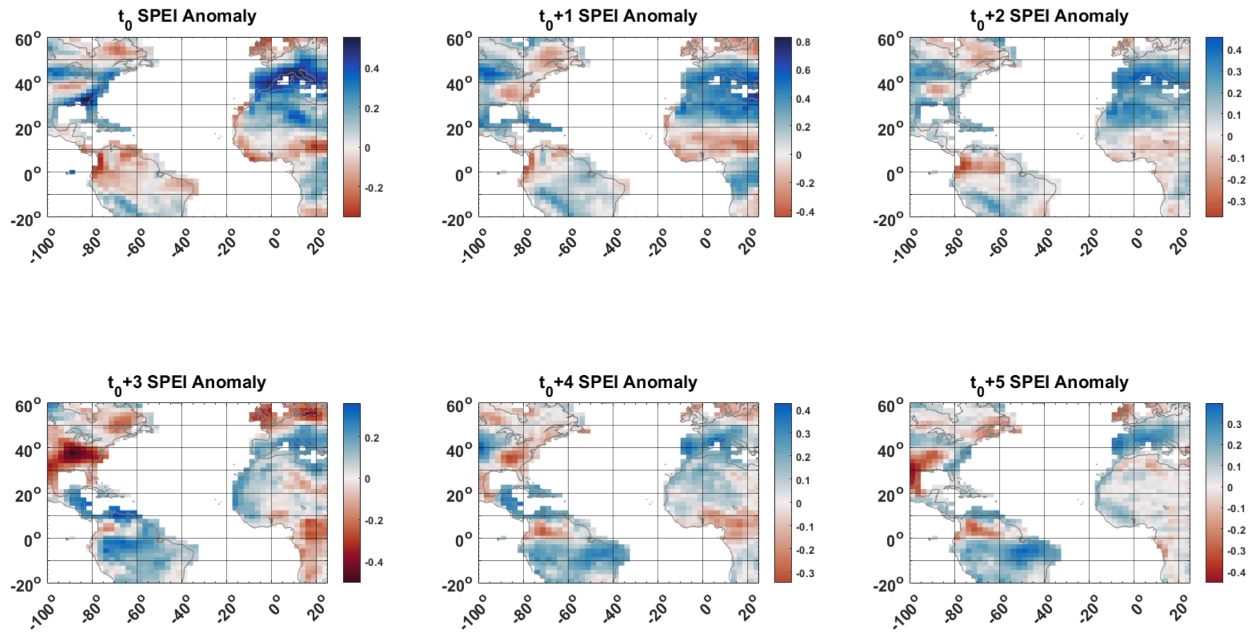

Fig. S19.

Composite SPEI anomalies during and up to five years after an eruption across the study period (1140–2000 CE). Each year in an eruption sequence was grouped by lag year (see Fig. 6e) and averaged before calculating the SPEI anomalies. SPEI values are from PHYDA, and anomalies were calculated relative to the gridded mean SPEI of the study period (1140–2000 CE). **(a)** Average SPEI anomaly during an eruption year (t_0). At t_0, the western flank is positioned toward the east (increasing BHI). **(b)** Average SPEI anomaly one year following an eruption (t_0+1), during which the western flank is positioned toward the west (decreasing BHI). **(c)** Average SPEI anomaly two years following an eruption (t_0+2), during which the western flank is positioned toward the west (decreasing BHI). **(d)** Average SPEI anomaly three years following an eruption (t_0+3), during which the western flank is positioned toward the west (decreasing BHI). **(e)** Average SPEI anomaly four years following an eruption (t_0+4), during which the western flank is positioned toward the west (decreasing BHI). **(f)** Average SPEI anomaly five years following an eruption (t_0+5), during which the western flank is positioned toward the east (increasing BHI).

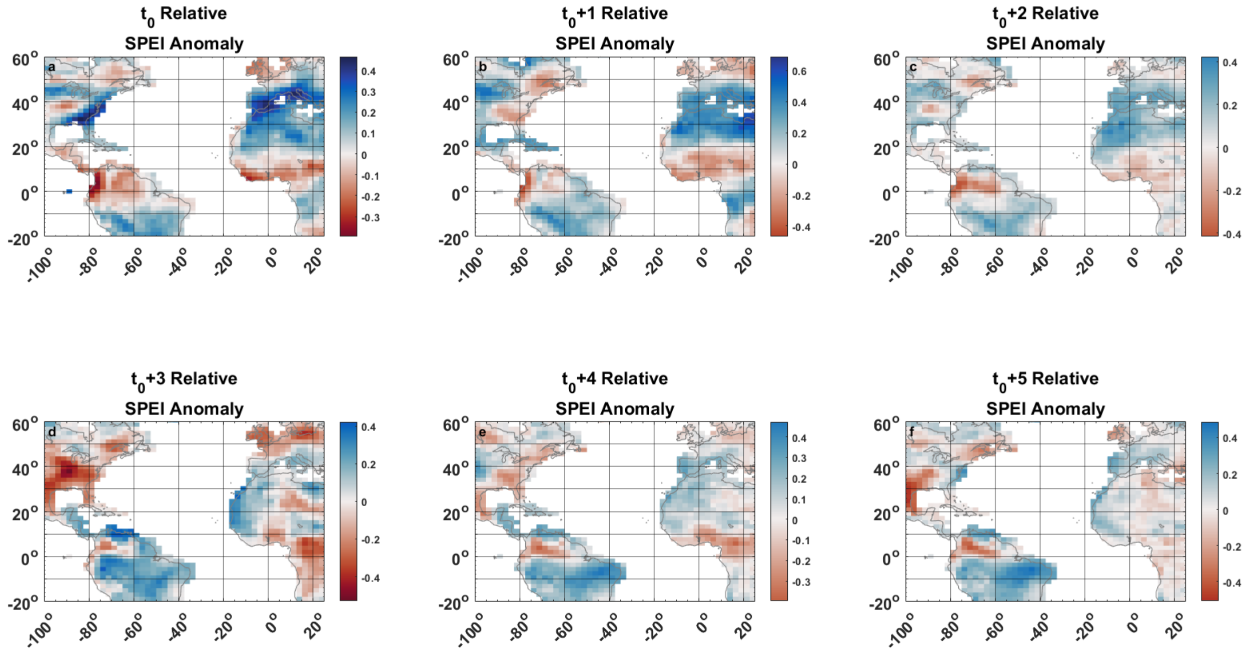

Fig. S20.

Composite relative SPEI anomalies during and up to five years after an eruption across the study period (1140–2000 CE). Each year in an eruption sequence was grouped by lag year (see Fig. 6e) and averaged before calculating the SPEI anomalies. SPEI values are from PHYDA, and anomalies were calculated relative to the gridded mean SPEI of the composite pre-eruption conditions (t_0-5 to t_0-1) **(a)** Average SPEI anomaly during an eruption year (t_0). At t_0, the western flank is positioned toward the east (increasing BHI). **(b)** Average SPEI anomaly one year following an eruption (t_0+1), during which the western flank is positioned toward the west (decreasing BHI). **(c)** Average SPEI anomaly two years following an eruption (t_0+2), during which the western flank is positioned toward the west (decreasing BHI). **(d)** Average SPEI anomaly three years following an eruption (t_0+3), during which the western flank is positioned toward the west (decreasing BHI). **(e)** Average SPEI anomaly four years following an eruption (t_0+4), during which the western flank is positioned toward the west (decreasing BHI). **(f)** Average SPEI anomaly five years following an eruption (t_0+5), during which the western flank is positioned toward the east (increasing BHI).

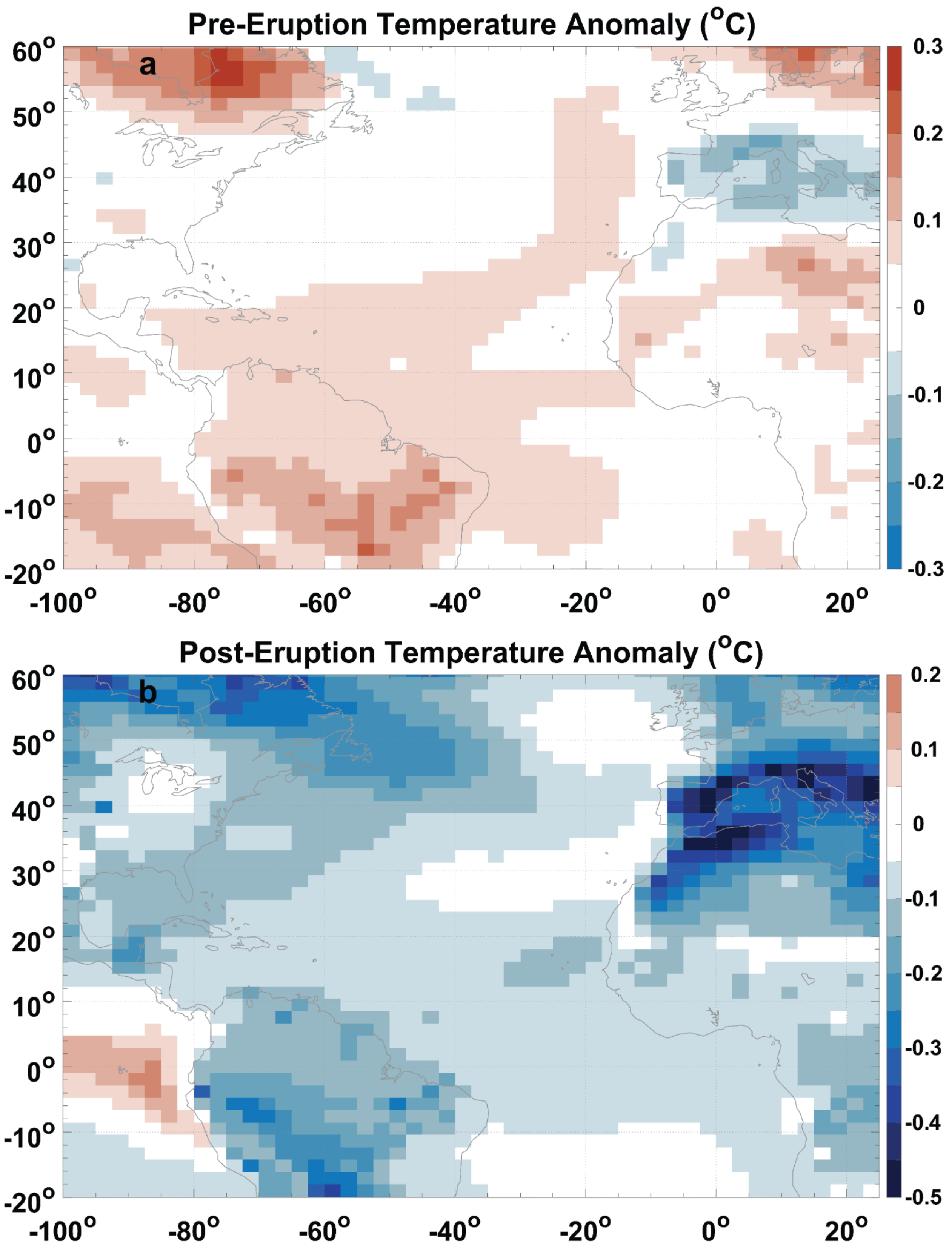
Pre-Eruption Temperature Anomaly (°C)
a
60°
50°
40°
30°
20°
10°
0°
-10°
-20°
-100°
-80°
-60°
-40°
-20°
0°
20°
0.3
0.2
0.1
0
-0.1
-0.2
-0.3
Post-Eruption Temperature Anomaly (°C)
b
0.2
0.1
0
-0.1
-0.2
-0.3
-0.4
-0.5

Fig. S21.

Mean pre- and post-eruption anomalies seen in 2-m temperatures (°C) from PHYDA. (a) Mean pre-eruption (t_0-5 to t_0-1) temperature anomalies (°C) relative to the study period mean (1140–2000 CE). **(b)** Mean post-eruption (t_0+1 to t_0+5) temperature anomalies (°C) relative to the study period mean (1140–2000 CE).

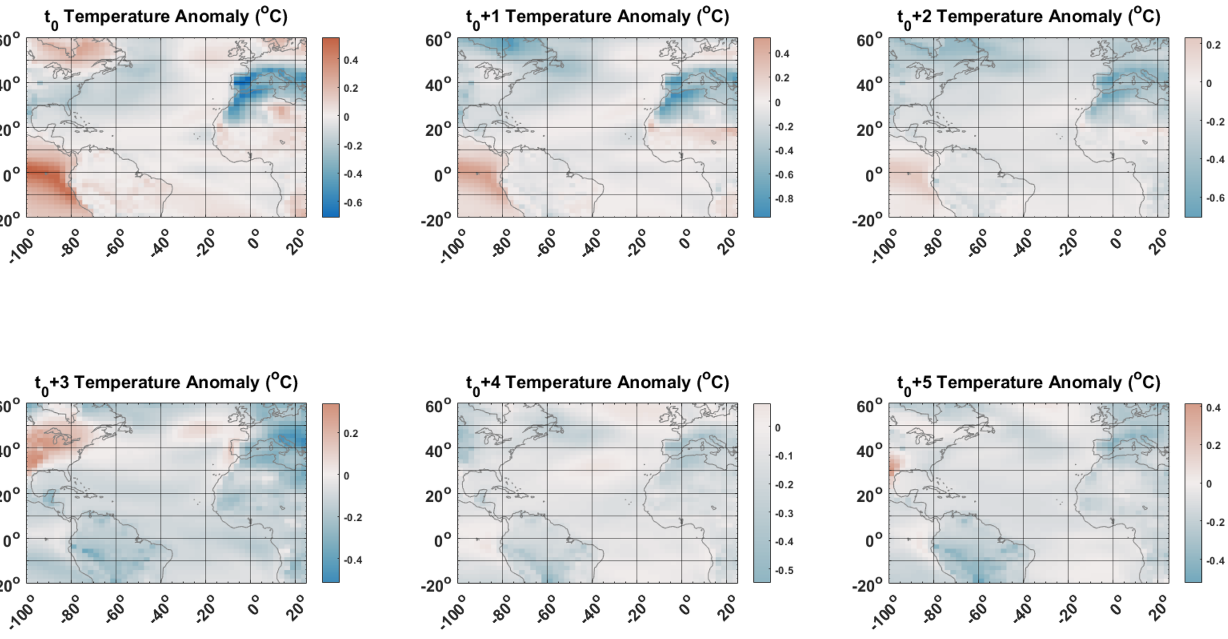

Fig. S22.

Composite 2-m temperature anomalies (°C) during and up to five years after an eruption across the study period (1140–2000 CE). Each year in an eruption sequence was grouped by lag year (see Fig. 6e) and averaged before calculating the temperature anomalies. Temperatures are from PHYDA, and anomalies were calculated relative to the gridded mean 2-m temperature of the study period (1140–2000 CE). **(a)** Average 2-m temperature anomaly (°C) during an eruption year (t_0). At t_0, the western flank is positioned toward the east (increasing BHI). **(b)** Average 2-m temperature anomaly (°C) one year following an eruption (t_0+1), during which the western flank is positioned toward the west (decreasing BHI). **(c)** Average 2-m temperature anomaly (°C) two years following an eruption (t_0+2), during which the western flank is positioned toward the west (decreasing BHI). **(d)** Average 2-m temperature anomaly (°C) three years following an eruption (t_0+3), during which the western flank is positioned toward the west (decreasing BHI). **(e)** Average 2-m temperature anomaly (°C) four years following an eruption (t_0+4), during which the western flank is positioned toward the west (decreasing BHI). **(f)** Average 2-m temperature anomaly (°C) five years following an eruption (t_0+5), during which the western flank is positioned toward the east (increasing BHI).

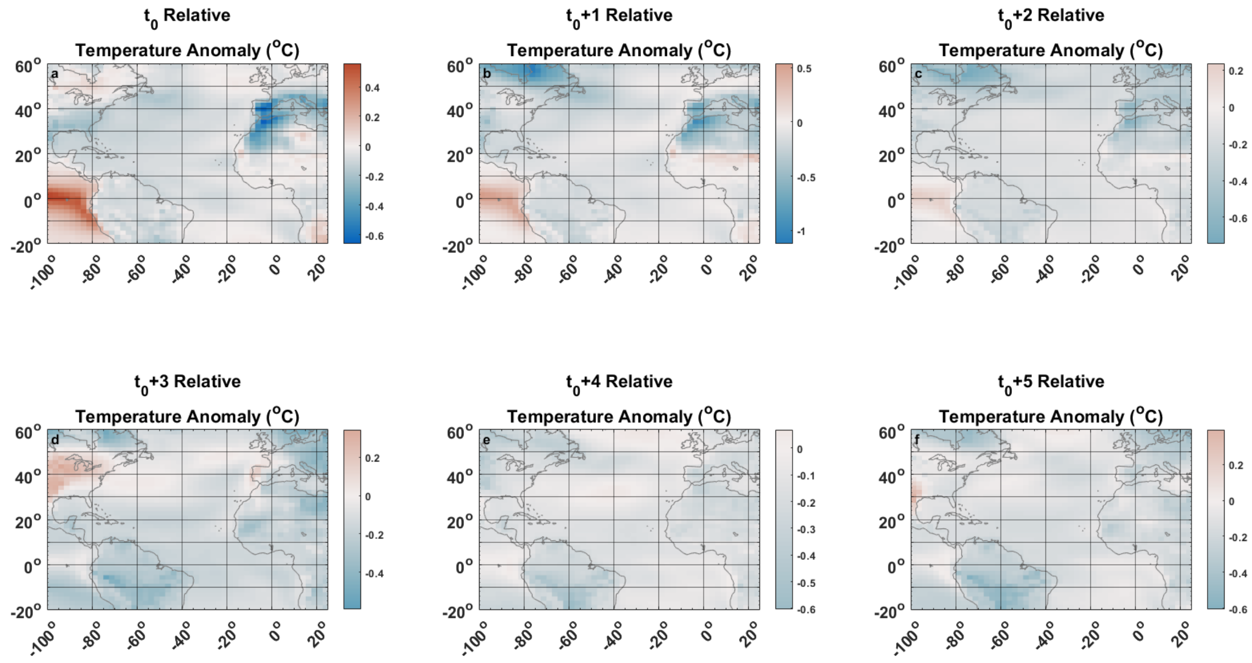

Fig. S23.

Composite relative 2-m temperature anomalies (°C) during and up to five years after an eruption across the study period (1140–2000 CE). Each year in an eruption sequence was grouped by lag year (see Fig. 6e) and averaged before calculating the temperature anomalies. Temperatures are from PHYDA, and anomalies were calculated relative to the gridded mean 2-m temperature of the composite pre-eruption conditions (t_0-5 to t_0-1). **(a)** Average 2-m temperature anomaly (°C) during an eruption year (t_0). At t_0, the western flank is positioned toward the east (increasing BHI). **(b)** Average 2-m temperature anomaly (°C) one year following an eruption (t_0+1), during which the western flank is positioned toward the west (decreasing BHI). **(c)** Average 2-m temperature anomaly (°C) two years following an eruption (t_0+2), during which the western flank is positioned toward the west (decreasing BHI). **(d)** Average 2-m temperature anomaly (°C) three years following an eruption (t_0+3), during which the western flank is positioned toward the west (decreasing BHI). **(e)** Average 2-m temperature anomaly (°C) four years following an eruption (t_0+4), during which the western flank is positioned toward the west (decreasing BHI). **(f)** Average 2-m temperature anomaly (°C) five years following an eruption (t_0+5), during which the western flank is positioned toward the east (increasing BHI).

Table S1.

List of 33 chronologies retained for the reconstruction model. Data are publicly available at the International Tree Ring Data Bank hosted by the National Centers for Environmental Information at the National Oceanic and Atmospheric Administration. Starred (*) entries of *Quercus montana* indicate datasets with species identified as *Quercus prinus*.

Site	Species	Lat.	Lon.	Years (CE)	Source
**Shot Beech Ridge**	*Quercus rubra*	35.60	-83.42	1771–2018	Ref. 93; this study (update)
**Scotts Gap**	*Liriodendron tulipifera*	35.59	-83.92	1684–2018	Ref. 94; this study (update)
**Amicalola**	*L. tulipifera*	34.34	-84.14	1552–2009	Ref. 95
**Bent Creek Experimental Forest**	*Q. alba*	35.30	-82.38	1733–1996	Ref. 95
**Bent Creek Experimental Forest**	*Q. spp.*	35.30	-82.38	1716–1996	Ref. 95
**Chattahoochee National Forest**	*L. tulipifera*	34.40	-84.16	1537–2009	Ref. 95
**Choctawhatchee River**	*Taxodium distichum*	30.28	-85.53	899–1992	Ref. 96
**Frick Creek**	*L. tulipifera*	34.46	-84.18	1537–2009	Ref. 95
**Frick Creek**	*Tsuga canadensis*	34.46	-84.18	1689–2009	Ref. 95
**Linville Gorge**	*Q. alba*	35.88	-81.93	1617–1977	Ref. 97
**Moody Tract**	*Q. lyrata*	31.56	-82.19	1803–1992	Ref. 98
**Ocmulgee River**	*T. distichum*	32.05	-83.30	1202–1984	Ref. 99
**Pine Mountain Combined**	*Q. montana*	32.50	-84.50	1794–2002	Ref. 95
**Stroubles Creek**	*Q. alba*	37.17	-80.48	1919–2006	Ref. 100
**Kelsey Tract**	*T. canadensis*	35.08	-83.18	1516–2018	Ref. 101; this study (update)
**Black River Update 2**	*T. distichum*	34.29	-78.11	0–2017	Ref. 102
**Bayou Deview**	*T. distichum*	34.95	-91.22	1133–1985	Ref. 103
**Georgia HWA**	*T. canadensis*	34.53	-84.39	1947–2011	Ref. 104

**New Hill Beaver Tree Farm**	*Pinus echinata*	35.40	-78.54	1879–1992	Ref. 105
**Pascagoula River**	*T. distichum*	30.35	-88.35	1466–1992	Ref. 106
**O'Leno State Park**	*Q. stellata*	29.55	-82.37	1852–1993	Ref. 107
**Suwannee River**	*Q. lyrata*	29.44	-82.57	1725–1994	Ref. 108
**Athens GA**	*P. echinata*	33.95	-82.32	1821–2004	Ref. 109; this study (update)
**Marshall Forest GA**	*Q. montana**	32.25	-85.19	1869–2008	Ref. 95
**Mammoth Cave**	*Q. alba*	37.18	-86.10	1649–2010	Ref. 110; this study (update)
**Pearl River**	*T. distichum*	32.87	-89.13	1546–1983	Ref. 111
**Hampton Hills**	*Q. alba*	35.49	-78.41	1770–1992	Ref. 112
**Altamaha River**	*T. distichum*	31.62	-81.80	929–1985	Ref. 113
**Lil Scaly Mountain**	*Q. alba*	35.02	-83.16	1599–2003	Ref. 95
**Kissimmee**	*P. palustris*	27.98	-81.22	1756–2005	Refs. 114, 115

Table S2.

List of volcanoes used in the superposed epoch analysis.

Year (CE)	Volcano	Location	VEI	Notes	Source
**1258**	Mount Samalas	Indonesia	7	Late 1257 or in 1258	Refs. 45, 90, 91
**1286**	Quilotoa	Ecuador	6		Ref. 45, 92
**1345**	El Chichón	Mexico	5		Refs. 45, 84, 85, 92
**1452**	Kuwae?	Vanuatu	6		Refs. 44, 45, 91
**1568**	Billy Mitchell	Papua-New Guinea	6		Ref. 44
**1600**	Huynaputina	Peru	6		Refs. 44, 45, 91, 92
**1640**	?	Indonesia	6		Refs. 44, 45
**1665**	Long Island	Papua-New Guinea	6		Ref. 44, 91
**1693**	?	?	?	1693: 6.7 Tg [S]<br>1695: 15.7 Tg [S]<br>Only 1693 is present in ice cores.	Refs. 44, 45
**1815**	Tambora	Indonesia	7		Refs. 44, 45, 91, 92
**1835**	Cosigüina	Nicaragua	5		Refs. 44, 45, 91, 92
**1884**	Krakatau (Kra-katoa)	Indonesia	6	Eruption Aug. 1883	Ref. 44, 91, 92
**1903**	Santa María	Guatemala	6	Eruption Oct. 1902	Ref. 44, 92
**1991**	Pinatubo	Philippines	6		Refs. 44, 45, 91, 92

Table S3.

Superposed epoch analysis results. Results were obtained using the “sea” function in the R-package “dplR”[81]. See Fig. 5e. 359

Lag Year	Mean BHI Departure	P-value
**0**	0.65	0.005
**1**	-0.81	0.001
**2**	-0.37	0.091
**3**	-0.35	0.097
**4**	-0.53	0.019
**5**	0.52	0.022

References S1. (separate file)

Tree ring and volcano dataset references.
Bregy_etal_BHIrecon_Dataset_References.xlsx
